# Ab Initio Molecular Dynamics Insights into Stress Corrosion Cracking and Dissolution of Metal Oxides

**DOI:** 10.3390/ma18030538

**Published:** 2025-01-24

**Authors:** Levi C. Felix, Qin-Kun Li, Evgeni S. Penev, Boris I. Yakobson

**Affiliations:** 1 Department of Materials Science & NanoEngineering, Rice University, Houston, TX 77005, USA; lf33@rice.edu (L.C.F.);; 2 Department of Chemistry, Rice University, Houston, TX 77005, USA

**Keywords:** hematite, corundum, stress corrosion cracking, dissolution, metadynamics, crack tip blunting

## Abstract

Oxide phases such as α-Fe_2_O_3_ (hematite) and α-Al_2_O_3_ (corundum) are highly insoluble in water; however, subcritical crack growth has been observed in humidity nonetheless. Chemically induced bond breaking at the crack tip appears unlikely due to sterically hindered molecular transport. The molecular mechanics of a crack in corundum with a reactive force field reveal minimal lattice trapping, leading to bond breaking before sufficient space opens for water transport. To address this, we model a pre-built blunt crack with space for H_2_O molecule adsorption at the tip and show that it reduces fracture toughness by lowering the critical *J*-integral. Then, we explore stress-enhanced dissolution to understand the mechanism of crack tip blunting in the oxide/water system. Density functional theory combined with metadynamics was employed to describe atomic dissolution from flat hematite and corundum surfaces in pure water. Strain accelerates dissolution, stabilizing intermediate states with broken bonds before full atom detachment, while the free energy profile of unstrained surfaces is almost monotonic. The atomistic calculations provided input for a kinetic model, predicting the shape evolution of a blunt crack tip, which displays three distinct regimes: (i) dissolution primarily away from the tip, (ii) enhanced blunting near but not at the apex, and (iii) sharpening near the apex. The transition between regimes occurs at a low strain, highlighting the critical role of water in the subcritical crack growth of oxide scales, with dissolution as the fundamental microscopic mechanism behind this process.

## 1. Introduction

The wide use of steel in power generation and carbon sequestration wells still needs to overcome many challenges as a result of its limited stability in prolonged exposure to the transport of environment fluids. Water is commonly present, either as the primary working fluid or as an impurity, such as in supercritical CO_2_ heat exchangers, where they can precipitate to the metallic/oxide surface and change the local pH [1,2], thus assisting corrosion in an autocatalytic way [3]. Alumina-forming austenitic alloys [4] have gained increasing interest over conventional stainless steel for applications such as heat exchangers [5] in power generation and supercritical/pressurized water reactors [6] due to their comparable cost and significantly superior corrosion resistance [7]. The latter is primarily due to the growth of a relatively thick layer of α-Al_2_O_3_ (corundum) between the alloy matrix and its oxide scale, which typically consists of spinel oxides with α-Fe_2_O_3_ particles and/or α-Cr_2_O_3_ layer [8]. One of the most detrimental effects of corrosion is chemically induced phase changes in the main alloy that generate internal stresses due to volume mismatch, which subsequently lead to cracks [9,10] in the oxide layer, exposing the underlying alloy to the working fluid and allowing continued corrosion of the matrix [11].

**Figure 1:**
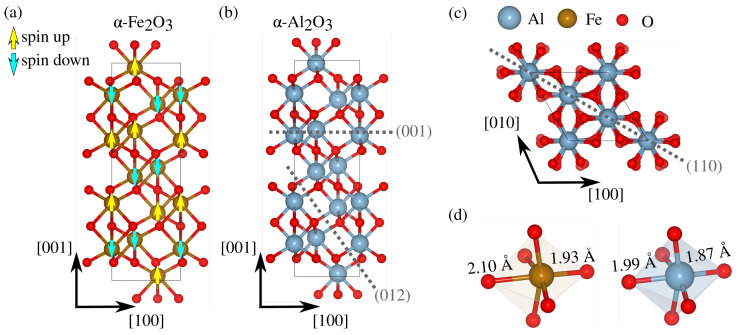
Crystal structure of (**a**) anti-ferromagnetic α-Fe_2_O_3_ and (**b**) α-Al_2_O_3_ with the (001) and (012) cleavage planes being labeled. (**c**) Top view of corundum (110) plane. (**d**) Octahedral units of both materials with long and short bond lengths, from our DFT calculations.

Another important aspect of the oxide/water interface is the fact that its strength can be severely reduced by crack propagation under subcritical conditions, as discussed in the early phenomenological studies [12] of the stress corrosion cracking (SCC) of silica (SiO_2_) glass and alumina ceramics (α-Al_2_O_3_) in water environments. A molecular mechanism of water splitting at a siloxane bridge (Si-O-Si) was proposed [13] for the observed reduced fracture toughness. The reaction steps of SCC are mainly determined by the covalent/ionic character of the cracked solid [14] and the sterically hindered channel size [15] for molecular transport promoting bond breaking at the crack tip. The strong covalence of SiO_2_ bonds favors stress-induced water dissociation. In contrast, mostly ionic solids display ionic solvation with non-dissociative chemisorption at the crack tip, as suggested by experiments on MgF_2_ [14]. Since it is well known that water dissociates even in stress-free materials with mixed ionic/covalent bonds, such as α-Al_2_O_3_ [16], the observed subcritical crack growth in single-crystal α-Al_2_O_3_ was also attributed to water dissociation at the crack tip [14,17,18]. Silica polymorphs possess relatively open structures due to the presence of Si-O-Si bridges, which makes it possible for small molecules, such as water, to diffuse to the crack tip and assist bond breaking by localized chemical reactions. Alumina polymorphs, on the other hand, lack such bridges in which tetrahedral O atoms are tightly packed with six-fold coordinated Al, as shown in Figure 1a. This question is then whether the same mechanisms of chemical rupture by reaction at the tip are involved in the water weakening of M_2_O_3_. Other works suggest that corrosion is initiated by local pitting induced by both metal dissolution [19] and local mechanical stresses [20,21], with kinetics controlled by solvent conditions (temperature, pH) and relative humidity [22]. After nucleation, a surface notch becomes a stress concentrator by geometrical changes induced by surface diffusion and/or evaporation leading to the Grinfeld instability of stressed solid surfaces [23]. This process can occur cyclically, eventually leading to catastrophic material failure [21,24]. Recent corrosion inhibition strategies are mostly focused on using polymeric coatings on carbon steel in water and carbon dioxide environments [25,26].

In this work, to investigate the subcritical crack growth behavior in metal oxides we first determine the lowest energy cleavage planes in α-Fe_2_O_3_ and α-Al_2_O_3_ with density functional theory. Then, due to the required large system sizes, we analyze the energetics of a crack tip in α-Al_2_O_3_ using classical force-field ReaxFF to evaluate the possibility of water-assisted propagation. The lack of space for molecule transport leads us to consider the water adsorption at the tip of a blunt (and wider) crack, where the *J*-integral quantifies the strength reduction. By proposing atomic dissolution as a necessary step for SCC, due to the complex nature of the whole blunting process, we consider strained flat surface/water interfaces to investigate the role of the mechanical stress of the atomistic steps of the ion free energy surface of dissolution using ab initio metadynamics. From the free energy barriers calculated, we parameterized an analytical kinetic model of macroscopic crack tip blunting.

## 2. Materials and Methods

### 2.1. Fracture Energies and Crack Tip Analysis

**Figure 2:**
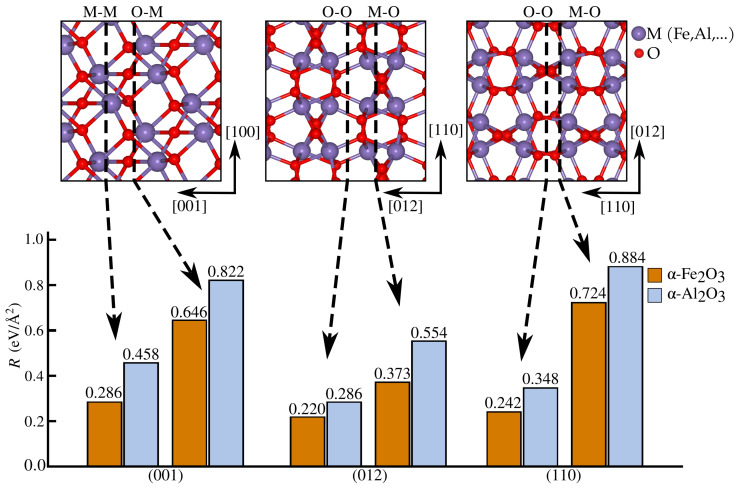
Fracture energy *R* values for all cleavage planes considered, computed with DFT. Each surface termination is marked by dashed line arrows pointing to their corresponding *R* values.

In order to determine the crystal orientation in subsequent crack propagation and atom dissolution, we search for the one with the lowest fracture toughness. For the three low-index orientations of α-Fe_2_O_3_ and α-Al_2_O_3_, two cleavage planes are selected in each, and their fracture energies are compared, defined as $R=\gamma_{1}+\gamma_{2}=\left(E_{\mathrm{slab}}-NE_{\mathrm{bulk}}\right)/A$, where $\gamma_{1}$ and $\gamma_{2}$ refer to the surface energy of each crack surface (which reduces to 2γ in Griffith’s criterion). $E_{\mathrm{slab}}$ is the total energy of a slab whose surfaces correspond to the cleavage planes indicated in Figure 1b,c, comprising *N* α-Al_2_O_3_ units and surface area *A*. The selected planes, (001), (012), and (110), are known to be the ones with the lowest surface energy obtained by first-principle investigations. They are also complemented by recently introduced simplistic analysis based on bond density on the cleavage plane under consideration [27]. All energies are calculated using density functional theory (DFT) with the CP2K software [28]. Exchange and correlation are described by the Perdew–Burke–Ernzerhof functional and for α-Fe_2_O_3_ the DFT+*U* formalism is adopted. Using U=4 eV results in a bandgap of bulk hematite closer to the experimental one. Additionally, we initially set an anti-ferromagnetic state with spins oriented along the (001) direction, as depicted in Figure 1a. All calculations are performed at the Γ point with a plane-wave cutoff of 500 Ry in conjunction with a DZVP basis set, along with the Goedecker–Teter–Hutter pseudopotentials. Dispersion interactions are included through Grimme’s D3 van der Waals corrections. Before building the slab models, a lattice optimization of 3×3×1 supercells is performed until all forces are smaller than $4.5\times 10^{-4}$ Ha/a_0_. Our DFT optimizations predict the well-known symmetry of the corundum lattice of both α-Fe_2_O_3_ and α-Al_2_O_3_, with two distinct Fe(Al)-O bond lengths near experimental values, as shown in Figure 1d. Slab thickness for all cleavage planes considered is the smallest one possible, but still larger than 10 Å, with a 10 Å vacuum in the normal direction ($\hat{z}$) to avoid spurious interactions between the two terminations. The two types of terminations analyzed can be categorized (Figure 2) as symmetric and asymmetric, including dipole corrections to avoid spurious effects from the different terminations.

**Figure 3:**
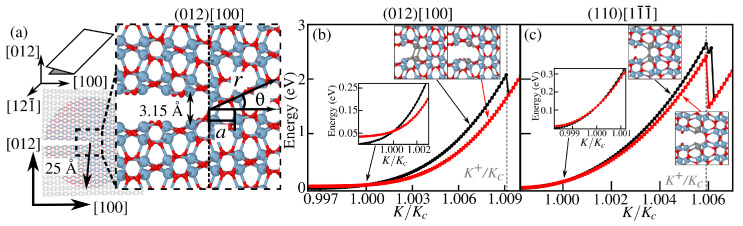
(**a**) Geometric setup for the crack tip analysis for α-Al_2_O_3_ corresponding to a (012) cleavage plane and [100] as the propagation direction (view along [12$\bar{1}$] where the gray region represents the frozen boundary and the colored (red and blue atoms) region is allowed to relax at every load step. Energy as a function of load *K* for crack tips centered at two consecutive bonds, states *A* and *B*, as illustrated in the insets, for (**b**) (012)[100] and (**c**) (110)[1 $\bar{1}$ $\bar{1}$] crack. An enlarged view of the region near the critical load $K_{c}$ is also shown in the inset. Atoms that comprise the crack tip are highlighted in light gray. ReaxFF-computed energies for state *A* are shown as black dots, while for state *B* are shown as red squares, with nearly a $E∼K^{2}$ behavior.

Crack tip studies were carried out along the lowest surface energy planes (012) and (110) of α-Al_2_O_3_, with the simulation setup being shown in Figure 3a. Due to the large size of crack geometries, Al-O-H interactions were modeled using ReaxFF [29], which describes well properties such as brittle fracture, elastic constants anisotropy, and surface hydrolysis. All ReaxFF calculations are performed using LAMMPS [30] combined with the ASE library [31]. To the best of our knowledge, no available force field predicts the brittle fracture of α-Fe_2_O_3_ reasonably. Thus, the methodologies employed in our work differ between different parts of the results, viz. figures. To clarify this point, we present a flowchart in the Supplementary Materials Section S1, which breaks down the methods used here. To focus only on the tip region, we first displaced all atoms according to the mode-I crack displacement field as follows:

(1) $$u=\left(u_{x},u_{y}\right)=\frac{K}{2Y^{\prime }}\sqrt{\frac{r}{2\pi }}\left(\kappa -\cos\theta \right)\left(\cos\frac{\theta }{2},\sin\frac{\theta }{2}\right)$$

where *K* is the stress intensity factor that characterizes the load state of the crack, $Y^{\prime }$ is the normalized Young’s modulus, Y/(1+ν), ν is the Poisson’s ratio, and κ=3−4ν for a plane strain problem (since the out-of-plane direction is kept fixed). The polar coordinates (*r*, θ) are measured from the crack tip, located at the first unbroken bond. With increasing load *K*, a circular region of 50 Å diameter, centered at r=0, is allowed to relax during geometry optimization while all other atoms, which are depicted in black in Figure 3a outside this circle, are fixed. Crack propagation occurs as the crack tip moves by one lattice parameter *a*. We first consider state *A*, where the crack tip is located at the center of the square region in Figure 3a. To obtain the next state, we apply the same *u*-field of Equation (1), but now shifted, to the center on the next unbroken bond, causing the bond at the center of the system to break. This results in crack propagation by *a* from state *A* to some new state $B^{\prime }$, while retaining *u* centered on a different bond. The final state is obtained by substituting the frozen atoms from the state *A* and performing geometry optimization to reach the fully relaxed state *B*. The energy change of both states *A* and *B* for cracks along (012) and (110) are shown in Figure 3b,c, respectively, as discussed in the next section. Such a procedure was applied in prior crack propagation studies, especially in the atomistics of brittle fracture of graphene [32,33].

### 2.2. *J*-Integral from Atomistic Simulations

To quantify the mechanical energy released upon bond breaking, we compute the so-called *J*-integral [34]. For a crack along the *x*-axis under load along *y*, *J* is given by closed contour integral:

(2) $$J=\int_{\Gamma }\left(w\delta_{ij}-\sigma_{ij}\;\frac{\partial u_{j}}{\partial x_{1}}\right)n_{j}\;d\Gamma =\oint_{\Gamma^{*}}\left(w\delta_{1j}-\sigma_{ij}\;\frac{\partial u_{j}}{\partial x_{1}}\right)qn_{j}\;d\Gamma$$

**Figure 4:**
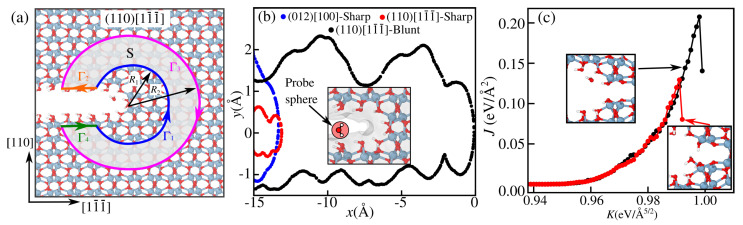
(**a**) Blunt tip model for a (110)[1 $\bar{1}$ $\bar{1}$] crack with passivated surfaces; the definition of the contour $\Gamma^{*}={⋃}_{i=1}^{4}\Gamma_{i}$, used to compute the *J*-integral, is illustrated, with arrows indicating the direction of traversing. (**b**) Solvent-accessible surface profiles for sharp cracks (012) [100] and [110][1 $\bar{1}$ $\bar{1}$], and a blunt (110)[1 $\bar{1}$ $\bar{1}$] crack. A probe sphere with a diameter of 2.6 Å was used to represent a water molecule, where the corresponding Connolly surface is shown in the inset for a blunt (110)[1 $\bar{1}$ $\bar{1}$] crack. (**c**) Computed *J*-integral for water-assisted cracking (red curve) compared with pure Al-O bond breaking (black curve).

For an applied load along the *y*-axis, the crack is expected to propagate along *x*, which is why the Kronecker delta $\delta_{1j}$ filters only the x component of the vector $n_{j}$ outward normal to the curve in the portion of the strain energy density *w*. The *j*-th component of the vector, the second term of Equation (2), comprises a summation of the index *i* of the ij-th component of the stress tensor $\sigma_{ii}$ and the displacement gradient $\partial u_{i}/\partial x$, with $u_{i}$ being the displacement along the *i* direction. The integration is over the contour $\Gamma^{*}$, as shown in Figure 4a. The weighting function q makes it possible to write *J* in a closed contour and should satisfy q=1 along $\Gamma_{1}$ and 0 along $\Gamma_{3}$. Along $\Gamma_{2}$ and $\Gamma_{4}$, the integrals should cancel out due to negligible stress along crack surfaces far enough from the tip (cohesive zone). For a circular contour, $q\left(r\right)=\left(r-R_{2}\right)/\left(R_{1}-R_{2}\right)$. From the divergence theorem, the *J*-integral is now given by the surface integral over the domain S:

(3) $$J={\;\int \int \;}_{S}\left(w\delta_{1j}-\sigma_{ij}\frac{\partial u_{i}}{\partial x_{1}}\right)\frac{\partial q}{\partial x_{j}}\;dS$$

We use the discrete form of Equation (3), which is more suitable for atomic systems since it includes a large area that tends to minimize the error due to the mismatch of the atomic domain shapes with the shapes in the continuous form, where the *q* function is used; it reads

(4) $$J=\frac{1}{L_{z}}\sum_{\alpha \in S}\left(w^{\alpha }\delta_{1j}-\sigma_{ij}^{\alpha }\frac{\partial u_{i}^{\alpha }}{\partial x_{1}}\right)\frac{\partial q^{\alpha }}{\partial x_{j}}V^{\alpha }$$

where the summation extends over all atoms α inside the domain *S*. Note that $w^{\alpha }V^{\alpha }$ and $\sigma_{ij}^{\alpha }V^{\alpha }$ are the energy and the atomic stress, respectively. Both quantities are reported by LAMMPS with no need to define an atomic volume. As explained in the Results and Discussion section, narrow sharp cracks hinder diffusion, inset in Figure 4b. For a water-assisted fracture, one thus needs to consider a blunt crack, as shown in Figure 4c. Both the strain energy and the displacement gradient are calculated from a reference state, which is the unstrained (K=0) blunt crack. Details on the definition of the displacement gradient for discrete systems are given in [35]. The *J*-integral form in Equation (4) has been successfully used in previous atomistic simulations of cracks [36,37] and dislocations [38], in contrast to continuum approaches previously considered [39,40,41,42]. At the end of Section 3.1, the reduction in *J* due to molecule adsorption at the tip apex is investigated.

Although there is a well-known relation from elasticity theory as $J=K^{2}/Y$, here, in this work, *K* is an input parameter that dictates crack opening and *J* is computed from the stress field around the crack tip. In general, as we will see in the Results and Discussion section, $J_{c}>K_{c}^{2}/Y$, where the exceeding mechanical energy released *J* may come from an overestimation of lattice trapping from adopted force field.

### 2.3. Free-Energy Sampling with Ab Initio Metadynamics

**Figure 5:**
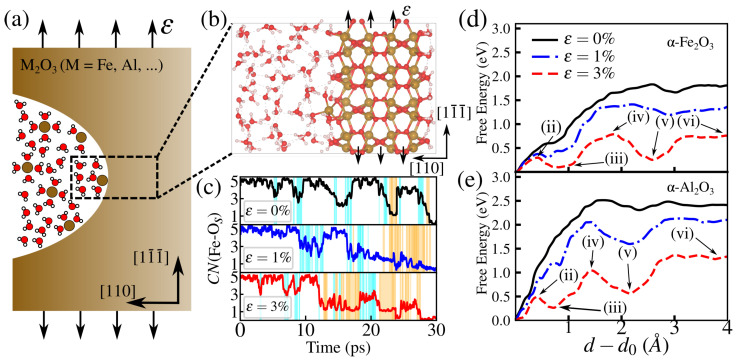
(**a**) Schematics of a crack blunted by atomic dissolution. (**b**) Zoomed view of the strained flat surface model used in the metadynamics simulations for the free energy of atom detachment from a (110) surface, with the reaction coordinate d indicated as the orthogonal distance of a surface atom to a plane determined by the position of three fixed atoms. (**c**) Time evolution of the coordination number of Fe with O atoms from the surface of oxide, CN(Fe-Os), (at strains ε = 0, 1, and 3%) keeping track of the bond-breaking events during the whole process. The free energy surfaces of metal atom dissolution for hematite (**d**) and corundum (**e**). Intermediate steps (ii)–(vi) are indicated on the free energy paths and are discussed in Figure 6a.

Although non-hydrous oxides such as α-Fe_2_O_3_ and α-Al_2_O_3_ are known to be insoluble in water, ion dissolution can be facilitated by surface defects such as step edges, kinks, and terraces [43], making the atomistics of crack blunting through dissolution complex multiscale task. To simplify it, we focus on how the ions dissolve from a flat surface. To this end, we examined how applied strain affects the free energy landscape, simulating the effect of far-field applied stress near a blunt crack tip, as shown in Figure 5a. The flat surface analysis is then used in Section 3.2 to predict changes in crack tip morphology due to dissolution. On top of each slab, 60 model water molecules were added using PACKMOL [44] in 10–12 Å so that nominal water density was reached in between the two ends of the slab. Since we aim to single out the role of water, complicating factors such as dissolved ionic species (e.g., Na^+^ or Cl^−^, in basic conditions) are not considered. Prior to equilibration with ab initio Born–Oppenheimer molecular dynamics (AIMD), a pre-equilibration step using the classical force field CLAYFF [45] was performed in LAMMPS for all water molecules with all atoms from the oxide and surface hydroxyl groups fixed to obtain a good starting point for AIMD runs. Production AIMD runs span 2 ps in the NVT ensemble at T=300 K, which is maintained through velocity rescaling. The deuterium mass (2 amu) is used for all hydrogen atoms, allowing the use of integration timestep of 1 fs. The free energy of water-assisted removal of surface atoms is sampled by metadynamics (MTD), using Gaussians 0.1 Å wide and $3\times 10^{-3}$ Ha high. The total time of MTD runs ranges from 30 to 60 ps, and they are stopped after the free energy surface remains unchanged as more Gaussian hills are added. The reaction coordinate is chosen as the distance *d* of a target Fe/Al from the plane defined by three (fixed) atoms in the middle of the slab, as shown in Figure 5b. A quadratic wall is placed below the target atom to avoid sampling regions of *d* much below its initial value which are not of interest to the present study. During the MTD trajectory, multiple Fe/Al-O bond-breaking events are registered by analyzing the coordination number CN of a target atom with nearby O atoms belonging to the oxide surface and bulk, being defined as

(5) $$CN=\sum_{i=1}^{M}\frac{1-{\left(\frac{r_{ij-d_{0}}}{r_{0}}\right)}^{n}}{1-{\left(\frac{r_{ij-d_{0}}}{r_{0}}\right)}^{m}}.$$

The summation is over all *M* atoms in the chosen group, which in this case includes each O atom from the oxide slab (O*_s_*), $r_{ij}$ is the distance between the target ion and O*_s_*, $r_{0}=1.5$ Å is the equilibrium distance. This switching function is shifted by $d_{0}=1.0$ Å, so that only water molecules closer than ∼3 Å from the target Al contribute to its CN. We set n=4 and m=10, as previously used in the study of atom dissolution from γ-Al_2_O_3_ in water [46].

## 3. Results and Discussion

### 3.1. Fracture Energy and Lattice Trapping of Cracks in α-Al_2_O_3_

As fracture toughness is governed by fracture energy *R*, crack propagation in crystalline brittle solids typically follows the lowest-*R* crystallographic planes, by Griffith’s criterion. We determined fracture energies in six cleavage planes, two for each low-index orientation considered, as shown in Figure 2. The symmetric O/O termination on the (012) plane had the lowest fracture energy among the surfaces studied, followed by the O/O termination on (110). A crack initially formed on (001) (see the Supplementary Materials, Figure S1) deflected onto the (012) plane, consistent with the experimental findings, showing stable crack growth on (012) and (110) and unstable growth on (001) in α-Al_2_O_3_ [47]. Previous DFT calculations [27,48] also identified (012) as the lowest energy surface in α-Fe_2_O_3_. Fracture energies in α-Fe_2_O_3_ are generally lower than in α-Al_2_O_3_ for the same cleavage planes, particularly (001), making iron oxides more susceptible to degradation after brittle fracture conditions are met. Using the geometric setup shown in Figure 3a, a simulation is initiated from an opened crack (as described in the Materials and Methods Section) with a (012) cleavage plane, propagating along [100], together denoted as (012)[100]. An initial load $K^{-}=0.996K_{c}$ is chosen, where $K_{c}=0.648$ eV Å^−5/2^ is the critical stress intensity factor. For load $K<K^{-}$, the crack tends to heal. The critical load $K_{c}$ is defined as the load where the energies of the initial (A) and final (B) states are equal. At $K^{+}=1.008K_{c}$, as shown in Figure 3b, the crack in state *A* spontaneously breaks and its configuration coincides with that of state *B*. This $K^{+}$ value defines the upper limit of the lattice trapping range. Beyond $K^{+}$, the crack is unstable at its initial position and propagates by *a*-shifts. Propagation in a perpendicular direction, [12$\bar{1}$] in Figure 3a, is associated with higher *K*, as shown in more detail in Figure S2.

The motion of a crack (110)[1 $\bar{1}$ $\bar{1}$], shown in Figure 3c, involves two types of atomic configurations alternating appearing at the crack tip: (1) two sets of two nearly vertical Al-O bonds (one set for state *A* and the other for state *B*), and (2) two tetrahedral O atoms forming an X-shaped configuration. The transition from *A* to *B* in Figure 3c involves a small energy change since stress relaxation is much more pronounced where fracture propagates from a crack tip of type (1) to (2) described above, with $K_{c}=0.938$ eV Å^−5/2^. The black curve, corresponding to state *A* of (110)[1 $\bar{1}$ $\bar{1}$] crack, shows a second peak very near $K^{+}$ that corresponds to state *B*, which is followed by a sudden drop to a state where the crack tip is located at an X-shaped configuration (the energetics of this crack tip type is described in the Supplementary Materials, shown in Figure S2).

The chemically assisted bond rupture in subcritical crack growth requires a molecule to reach an intact bond and react to break it. However, even at the maximum load $K^{+}$, the crack tip opening is only ≃3.15 Å, comparable to the diameter of a water molecule (≃2.6 Å). This space may be further reduced by water or environmental passivation of the exposed crack surfaces. To assess this, we calculated the accessible solvent area through the so-called Connolly surface [49] (commonly used in biomolecular studies). Details of its calculation are provided in the Supplementary Materials in Section S3. Such an approach helps us to determine whether H_2_O can access and react with unbroken bonds at the crack tip, clarifying the feasibility of chemically assisted bond rupture.

As an example, we consider the blunt (110)[1 $\bar{1}$ $\bar{1}$] crack in Figure 4a, where two central rows of Al and four O atoms are removed, making space for molecules to react with the tip, in a way that leaves with a ratio of 3/2 of O and Al number of atoms to keep the overall stoichiometry of the system (to avoid any charge accumulation and change of the oxidation states +3 and −4 for Al and O, respectively). Such material removal results in unsaturated Al and O atoms, which are passivated by OH groups and H atoms, respectively, such that they recover their coordination in the bulk, making them unreactive. This allows us to isolate the effect of strain on the chemo-rupture by water. The surface profiles of the sharp cracks along (012)[110] and (110)[1 $\bar{1}$ $\bar{1}$], the same as in Figure 3, reveal no available space near the tip at x=0 but only as far as ∼12 Å away, as shown in Figure 4b. For the blunt tip, considerable space is accessible near the tip, even in the presence of passivation.

For a comparison, we also computed the Connolly surface for cracks in a silica polymorph and found no need for a blunt crack to permit the kinetics of water-assisted cracking; such analysis using the nudged elastic band (NEB) method is detailed in the Supplementary Materials (Figures S3 and S4). This suggests that water can actively participate in crack tip chemistry, promoting bond rupture in silica even in sharp cracks, where straight Si-O-Si bridges facilitate bond breaking by dissociative chemisorption of the water molecule. As strain increases, such a process becomes more energetically favorable providing a larger driving force for subcritical crack propagation by one lattice parameter. The successive reaction steps of molecule dissociation near the tip are known to be stress-dependent [50] and only a single case is considered here, namely, where O from water adsorbs at the Si site at the tip followed by proton transfer from molecule to O on the Si-O-Si bridge. This mechanism; however, is absent in α-Al_2_O_3_ due to its more compact atomic structure. In the case of a blunt crack tip, Figure 4a, all ions remain chemically inactive, and a reaction could only be triggered by the presence of strained bonds, or a high-pressure environment forcing water adsorption near the tip.

In a purely mechanical bond-breaking process, the energy released from the applied load is converted into surface energy as the crack advances, as long as thermal dissipation through lattice vibrations and any other mechanisms are neglected. When the process is chemically assisted, less mechanical energy is needed since part of the energy to open the crack comes from the exothermic adsorption of molecules. To illustrate this, we used a blunt crack model in Figure 4a with a water molecule placed near the crack tip. The load was gradually increased until bond breaking occurred. Two scenarios were tested: (i) with the water molecule placed as-is and (ii) with constrained minimization, where a linear spring (spring constant k≃2 eV/Å^2^) was applied between the water’s oxygen atom and an aluminum atom at the tip apex. For a small *k* value, the behavior was similar to case (i), while a very large *k* caused an undesired effect by artificially displacing the aluminum atom. Using k≃2 eV/Å^2^ ensured that the water molecule was guided toward the tip without distorting the system. For water-assisted cracking in α-Al_2_O_3_, we computed the *J*-integral (see Section 2.3) for the two cases described previously, as shown Figure 4c. In the unconstrained case (i) above, where the water molecule is only held by intermolecular forces to the nearby OH passivating groups near the tip apex (black curve), the critical value $J_{c}$ is slightly larger than the fracture energy of a (110) cleavage with O/O termination, as shown Figure 1b, which corresponds to a “dry” fracture, since a pure bond breaking is involved in the crack extension at K≃1 eV/Å^5/2^ and $J_{c}=0.20$ eV/Å^5/2^. In the case of constrained minimization (red curve), the reaction is favored already at K≃0.99 eV/Å^5/2^ and $J_{c}=0.14$ eV/Å^5/2^, being nearly 50% lower. Such a reduction is comparable to the case of silica (as shown in Figure S5), only with the exception that water dissociation at the tip is observed, according to the Michalske–Freiman mechanism [13], contrasting the non-dissociative chemisorption in α-Al_2_O_3_.

### 3.2. Stress-Enhanced Dissolution

The limited molecule transport to the crack tip suggests that an alternative mechanism, likely involving crack tip blunting, might be operative where the molecule plays an active role in SCC. As dislocation-mediated plasticity is unlikely under typical conditions, appearing only at a very high *T* near the brittle-to-ductile transition, we explore dissolution as a plausible blunting process. Dissolution has been observed in other Fe and Al hydroxides [43,51,52,53,54], offering insight into how material removal reshapes the crack tip. An important step in this process is the removal/dissolution of a single surface atom. The energetics of this step are quantified here by mapping the free energy landscape of dissolution under applied strain, which mimics the stress environment at the crack tip, by employing ab initio metadynamics. We focus on dissolution near the tip region, as illustrated in Figure 5a. To represent this scenario, we model the dissolution process using a strained flat (110) surface, orthogonal to (012), which has the lowest energy; a crack in the (012) cleavage plane is maintained by tension perpendicular to [110]. As Gaussian-shaped hills are added as a bias potential, the system shown in Figure 5b will explore larger values of the reaction coordinate *d*, where the target atom is gently pulled from the surface. To analyze the effect of strain in such a process, we inspect the time evolution of the coordination number CN(Fe-Os) of hematite and the free energy paths of both oxides, shown in Figure 5c–e. As the strain increases, the time that the target atom spends with lower coordination increases. For instance, at ε=1%, CN(Fe-Os) oscillates between 5 and 1, at 5 to 20 ps, until it reaches stability in such a state with already four broken bonds, as the one shown in Figure 5c. Overall, the system resides longer in lower coordination number states as the strain increases. This is reflected in the free energy surface changes in Figure 5d for hematite. The small plateau at $d-d_{0}≃0.6$ Å turns into a local minimum. Such an effect is also observed in a second minimum at $d-d_{0}≃2.5$ Å for hematite and ≃2.1 Å for corundum.

Corundum exhibits similar behavior but with higher free energy barriers, as shown in Figure 5e. In the strain-free case, the free energy plateau reaches approximately 2.5 eV. A notable difference is the reaction coordinate $d-d_{0}$ corresponding to the second minimum, which occurs at 2.1 Å. This shorter distance compared to hematite is due to the shorter equilibrium Al-O bond length, which is generally smaller than Fe-O bonds.

**Figure 6:**
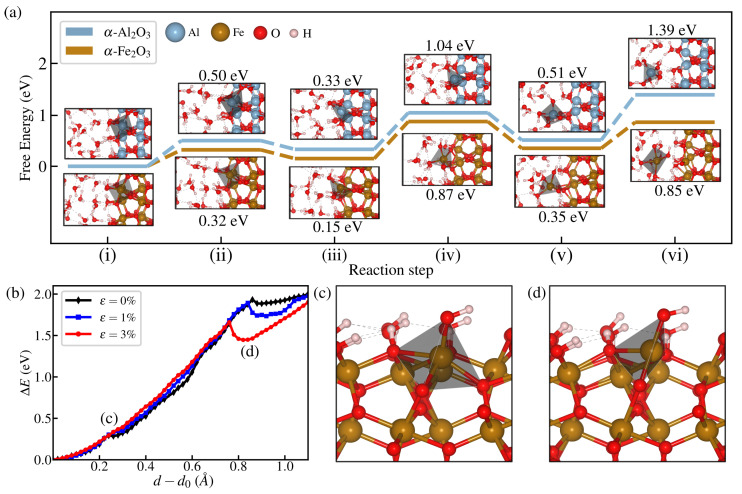
(**a**) Free energy diagram of the dissolution process at ε = 3% for both hematite and corundum. Reaction steps (i) and (vi) correspond to states where $d-d_{0}=0$ and 4, respectively, whereas intermediate ones are indicated in Figure 5d,e. (**b**) Energy change ΔE of static change of atom height d, where a geometry minimization is performed every step with the constraint of *z*-coordinate of the target atom. (**c**) The intermediate state where the bond below the target atom (here indicated by the coordination polyhedral) shows a small change in the curve in (**b**). (**d**) Lateral bond breaking presents a larger effect in the structural relaxation upon breaking, as observed by the deeper minima that arise from the increase in externally applied strain nearly parallel to such bond.

Several intermediate states are revealed from the free energy surfaces of both materials for 3% strain, where each configuration is shown in the reaction diagrams of Figure 6a. As can be seen, dissolution takes place by successive Fe(Al)-O bond-breaking events between the surface atom and oxygen from the oxide slab (Os) and is quantitatively monitored by the time evolution of CN, as shown in Figure 5c. The target surface atom is initially fully saturated with a six-fold coordination with O atoms, as indicated by its octahedral configuration in (i), where five of these bonds belong to the oxide and one to a passivating OH group, such that initially CN=5. In the first dissolution step at (ii) ($d-d_{0}≃0.4$ Å), the Fe(Al)-O bond directly below the target atom breaks, transitioning to a pyramidal configuration leading to CN=4. Then, small d increments yield the next bond-breaking events at $d-d_{0}≃0.6Å$ in (iii) and $d-d_{0}≃0.7$ Å at (iv). The next breaking leads to a state with a considerably higher distance with a single intact bond with the surface (CN=1) and forming a tetrahedral configuration with three additional Al-O bonds with oxygen from water. At this point, additional increments in d lead to a fully dissolved Fe^3+^ (or Al^3+^) ion, as shown in (vi). Apart from the evident effect of strain on reducing the free energy barrier for dissolution, another notable feature is the appearance of local minima before complete dissolution. y path can be explained by analyzing the change in energy when a single atom is statically pulled, where the whole structure is under different applied strains. As shown in Figure 6b, two bond-breaking steps are observed for all strains considered, but only the second breaking has a local minimum considerably deepened. Such a case corresponds to breaking a bond nearly parallel to the direction of applied strain. In a sense, this case is similar to a strain-driven stabilization of broken bonds in carbon nanotubes, where breaking bonds aligned with the load directions is the preferred breaking path for nanotube fracture [55].

Based on the results for the flat surface, we analyze how such a process can be translated to the blunting of a crack tip, adopting the approach of [56]. The complete crack blunting can be seen as a four-step process: (1) transport of water to the tip, (2) subsequent adsorption at the cavity surface near the tip, (3) metal atom detachment by dissolution reactions with nearby water molecules, and (4) ion diffusion away from the tip apex. The rate-limiting process is considered to be (3), since (1) and (2) occur spontaneously on metal oxide surfaces. As long as the applied strain is still within the elastic regime (σ=Yε, with *Y* being Young’s modulus of α-M_2_O_3_), the stress dependence of the free energy barrier (F) of atom removal can be considered up to first-order terms:

(6) $$F\left(\sigma \right)=F_{0}-V^{*}\sigma +O\left(\sigma^{2}\right),$$

where $F_{0}$ is the free energy barrier of the stress-free flat surface, σ is the applied stress, and $V^{*}$ is the activation volume, which gives the slope of F(σ) as a function of strain. Values of $V^{*}$ for both hematite and corundum are obtained from our metadynamics results and are reported in Table 1. Step (4) is facilitated by the chemical potential difference between the dissolved ions and the solid metal oxide, which changes considerably from a flat surface to a cavity. From the considerations above, one can obtain the rate of dissolution on a stressed, blunt cavity relative to an unstressed flat surface from transition state theory, as

(7) $$\frac{v}{v_{0}}=\exp\left(-\frac{\Delta F-\Delta \mu }{k_{B}T}\right),$$

where *v* ($v_{0}$) is the rate corresponding to a stressed blunt cavity (unstressed flat surface), $k_{B}$ is the Boltzmann constant, *T* is the temperature, and $\Delta F=F-F_{0}$, from Equation (6). The change in the chemical potential of the water/oxide surface Δμ promotes an overall reduction in the barrier of dissolution, which is further increased by the presence of both applied stress and local curvature of the crack. For an ellipsoidal shape [56], $\Delta \mu =V_{m}\left(\sigma^{2}/2Y-\gamma \kappa \right)$, where $V_{m}$ is the molar volume, γ is the surface energy and κ is the curvature. To describe changes in the crack shape, we need to evaluate the angular dependence of the corrosion rate (relative to the unstressed flat surface). In this way, we express the kinetic change in the crack shape by

(8) $$k_{B}T\ln\left(\frac{v}{v_{0}}\right)=V^{*}\sigma +\frac{V_{m}}{2Y}\sigma^{2}-\gamma V_{m}\kappa .$$

The explicit angular dependence follows closely the approach from [56] and, for completeness, is presented in the Supplementary Materials.

**Figure 7:**
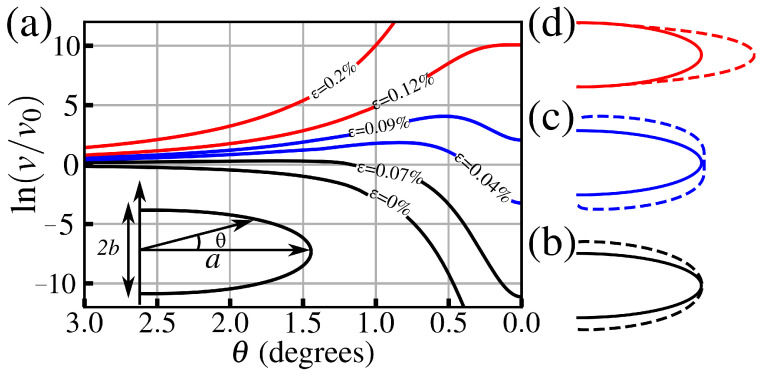
(**a**) Angular dependence of the dissolution rate *v* near the tip determined from Equation (8), with parameters corresponding to the α-Fe_2_O_3_ (110) surface. Each curve corresponds to a different strain value (as labeled). The *v* values are normalized by stress-free corrosion of a flat surface, $v_{0}$ (dashed line). The inset shows the tip geometry. Schematics of the crack tip blunting evolution are shown with steps consisting of (**b**) gross blunting, (**c**) enhanced tip blunting, and (**d**) gross sharpening.

Figure 7a shows the calculated v(θ) dependence for different applied stresses σ (normalized by the material’s tensile strength). As one increases the applied stress, it can be seen that the overall shape of v(θ) has three different behaviors characterizing three well-defined blunting regimes. For low σ values, the rate is minimum near the tip apex (small) and then increases for larger angles, which can be seen as gross blunting of the cavity where enhanced dissolution only takes place away from the apex, as shown in Figure 7b. As σ increases, the curves start presenting a peak near the apex and a local minimum in its vicinity, which represents an enhanced blunting near the tip apex, as can be seen in Figure 7c. As the load increases even further, a large peak emerges without any local minima nearby, representing a gross sharpening shown in Figure 7d, which corresponds to the last stage of the subcritical crack growth by water weakening. At this stage, the increase in crack length is accelerated, leading to a brittle fracture of the material. Such behavior is less commonly observed in silica glass, where a crack tip necking usually precedes enhanced corrosion at the tip apex [56].

We are aware that considerable dissolution may also be occurring on the crack surfaces far from the tip apex, but it does not contribute to crack extension and, therefore, is not considered in our analysis. It should also be noted that, due to the high anisotropy of α-Fe_2_O_3_, our conclusions are only valid for small angles around the tip apex.

## 4. Conclusions

This work explores water-assisted subcritical crack growth mechanisms in Fe and Al oxides. We found that chemical reactions at the tip of a sharp crack in Al_2_O_3_, unlike in silica polymorphs, do not follow the Michalske–Freiman mechanism [13] of water splitting and surface passivation. This led us to investigate crack tip blunting by atom dissolution. The effect of applied strain on the free energy of dissolution, computed using ab initio metadynamics, revealed two key trends: (i) a reduction in the free energy barrier with increasing strain and (ii) the emergence of two intermediate local minima due to strain-induced stabilization of broken bond configurations by stress relaxation. Crack tip shape evolution under strain, modeled using kinetic equations parametrized by first-principle data, revealed three stages of blunting: (i) increased dissolution away from the tip, (ii) enhanced blunting near the apex, and (iii) increased sharpening.

While the oxide/water interface has been widely studied, the role of water in subcritical crack growth of iron and aluminum oxide polymorphs remains underexplored. Our work reveals the atomistic details of this failure mechanism, which can lead to catastrophic failure of mechanical components under stress and aqueous environments. Beyond the free energy of dissolution, the slope of its strain dependence (activation volume, $V^{*}$) is critical for designing oxide scales resistant to subcritical crack growth, as it determines the stress required for transitions between blunting stages [56]. Such an effect would be characterized as intragranular cracking, which has been observed in experiments in the context of pipe corrosion in nuclear power plants [57]. Another relevant element of our modeling is that we deal with plane cracks. Recent work has observed one-, two- and three-dimensional crack propagation as a consequence of corrosion in metals [58].

This study focused specifically the effects of strain on dissolution. Since we observed relatively high barriers (up to 2.5 eV), it is important to note that structural defects—such as kinks and step edges, which often present in these materials—can facilitate dissolution by reducing the coordination of surface atoms. Investigating the role of such defects would require larger systems and a reparametrized force field to capture chemical changes accurately, which remains to be studied in the future. These findings are particularly relevant for Fe and Al oxides, common oxidation products of steel [11], and alumina-forming alloys used in energy systems for fluid transport, such as supercritical water and CO_2_ environments. Our results support an additional mechanism of subcritical failure in otherwise protective oxide scales.

Although many works focus on alumina/water interfaces, there is a limited amount of articles that have investigated the role of water in the subcritical crack growth of iron and aluminum oxide polymorphs. Atomistic modeling of stress corrosion cracking has previously focused on simplified model systems, such as surfaces and nanorods [50,59], which fail to account for the restricted fluid transport pathways characteristic of crack tip geometries. Specifically, heuristic arguments regarding the molecular mechanisms of water adsorption at metal oxide crack tips [14] encounter challenges when compared with our findings on the limited range of lattice trapping, where bond breaking can occur before water adsorption at the tip. Previous first-principle investigations have primarily addressed the transient behavior of corrosion during its initial stages [3]. In contrast, our study integrates both ab initio simulations and a macroscopic kinetic model to capture longer-term corrosion behavior. In this sense, our work reveals the atomistic details of the main mechanism of such a process, which can lead to catastrophic failure of mechanical parts under stress and exposure to an aqueous environment.

## Figures and Tables

**Table 1:** Free energy barriers *F* (in eV) and activation volume $V^{*}$ for all strain values considered in Figure 5d,e.

	F (ε=0%)	F (ε=1%)	F (ε=1%)	$V^{*}\;\left({Å}^{3}\right)$
Fe_2_O_3_	1.89	1.45	0.87	32.72
Al_2_O_3_	2.51	2.23	1.39	16.75

## Data Availability

Data supporting the conclusions of this work is available from the authors upon reasonable request.

## Supplementary Materials

The following supporting information can be downloaded at: https://www.mdpi.com/article/10.3390/ma18030538/s1, Figure S1: Crack tip evolution for hematite using different ReaxFF parameter sets, Figure S2: Crack tip evolution for corundum using different ReaxFF parameter sets, Figure S3: Free energy of aluminum dissolution computed with ReaxFF, Figure S4: Convergence of the free energy path and the free energy barrier of aluminum dissolution, Figure S5: Flowchart diagram summarizing the methodology used in this work, Figure S6: Crack on (001) plane with Al termination, Figure S7: Energy versus *K* for $\left(012\right)\left[12\bar{1}\right]$ and $\left(110\right)\left[1\bar{1}\bar{1}\right]$ cracks in corundum, Figure S8: Accessible solvent area measured by the Connolly surface with the profiles for β-cristobalite and corundum, Figure S9: Energetics of a sharp crack in a crystalline silica model, Figure S10: Kinetics of a sharp crack in cristobalite, Figure S11: *J*-integral calculation for the rupture and chemo-rupture processes involved in the fracture of cristobalite, Table S1: Comparison of lattice constants and bulk modu-lus for hematite for DFT and different ReaxFF parametrizations, Table S2: Comparison of lattice constants and bulk modu-lus for hematite for DFT and different ReaxFF parametrizations, Table S3: Fracture energies (R=2γ) corresponding to identical surface terminations of hematite as obtained by our results and other computational studies in the literature. References [12,13,14,15,17,27,29,30,31,32,37,43,46,48,50,54,56,60,61,62,63,64,65,66,67,68,69,70,71,72,73,74,75] are cited in Supplementary file.
