# Supplementary material for: Ab Initio Molecular Dynamics Insights into Stress Corrosion Cracking and Dissolution of Metal Oxides
Source: Materials (Basel). 2025 Jan 24;18(3):538. doi: 10.3390/ma18030538 (PMC11818312; doi:10.3390/ma18030538)
Supplement: Supplementary file 1 [file materials-18-00538-s001.zip › materials-3412799-supplementary.pdf]

SUPPLEMENTARY MATERIAL:

Ab Initio Molecular Dynamics Insights into Stress Corrosion Cracking and Dissolution of Metal Oxides

Levi C. Felix[1], Qin-Kun Li[1], Evgeni S. Penev[1], and Boris I. Yakobson[1,2]

[1]Department of Materials Science & NanoEngineering, Rice University, Houston, TX 77005, United States
[2]Department of Chemistry, Rice University, Houston, TX 77005, United States

S1 Further Details on Methodology

S1.1 Choice of Force Field

As of our initial goal, we aimed to study how stress corrosion cracking would occur in metal oxides such as hematite and corundum in a similar approach of how it happens in silica polymorphs (to be discussed in details on section S3), where a single molecule goes through the narrow channel of the crack and reaches the tip where chemical reaction occurs, thus, promoting sub-critical crack growth. The large sizes involved in crack propagation studies usually leads to the use of computationally affordable force fields. For our purpose (hematite/corundum interacting with water) we would need, ideally, a reactive force field with parameter set that includes Fe, Al, O and H. To our knowledge, only ReaxFF have been parametrized for the desired collection of chemical species. As we known that both materials present brittle fracture, we performed crack opening tests using all parameter sets available in the literature. For a reasonable brittle fracture behavior we should have a few requirements to select the force field, which include:

- absence of plasticity during crack propagation, such as tip amorphization or dislocation emission (for more details on these two effects, see the work of Huang et al. [1];
- reasonable estimate of the lattice trapping effect, which gives the range of applied load necessary to heal or propagate the crack [2];
- reasonable estimate of the elastic constants of the crystal.

Table S1: Comparison of lattice constants and bulk modulus for hematite for DFT and different ReaxFF parametrizations.

Method	a/b (Å)	c (Å)	B (GPa)
DFT	5.04	13.77	205
Aryanpour et al.	4.89	12.61	210
Shin et al.	4.99	11.94	293
Huang et al.	4.97	14.94	49318

For FeOH, we have tested three main parameter sets: Aryanpour et al. [3], Shin et al. [4] and Huang et al. [5] The first two cases produce strong tip amorphization before crack opening, as can be seen in Figure S1(a) and (b). However, they present reasonable elasticity, here measured by the bulk modulus B and, shown in Table S1. The more recent ReaxFF version provided by Huang et al. shows good brittle fracture behavior, as shown in Figure S1(c), however, it overestimates B by two orders of magnitude, as shown in Table S1. Such a strong Fe-O interaction may result in the large lattice trapping where the crack can be opened considerably before the tip bond breaks. In Figure S1, atoms are colored by the number of neighbors, where nanoplasticity (in this case, amorphization) at the tip can be seen as changes in the coordination number (CN) by atoms near the tip but still in the bulk region. Note that, initially in the bulk CN(M)=6 and CN(O)=4, whereas in the surface, along the (012) cleavage plane, CN(M)=5 and CN(O)=3, where M is either Fe or Al.

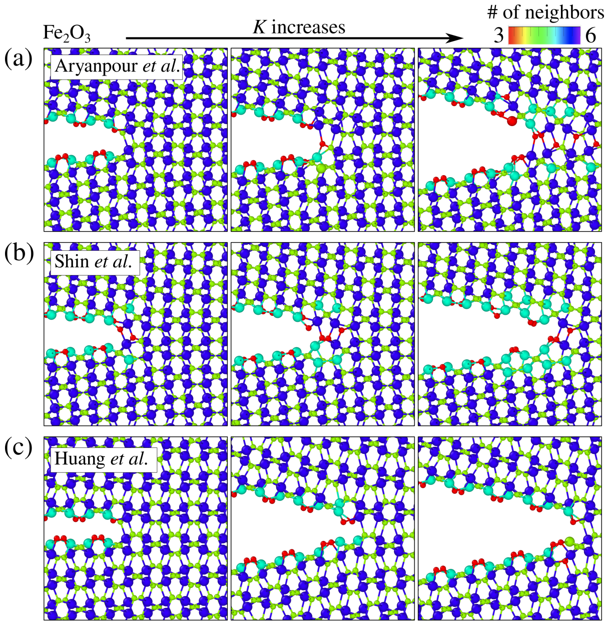

Figure S1: Crack tip evolution for hematite using different ReaxFF parameter sets: (a) Aryanpour et al. [3], (b) Shin et al. [4] and (c) Huang et al. [5]. Atoms are colored by the number of first neighbors.

For AlOH, we have also tested three parameter sets: Sen et al. [6], Hong et al. [7] and Zhang et al. [8] The first two also present tip amorphization, while the most recent ReaxFF version of Zhang et al. presents good brittle frac-

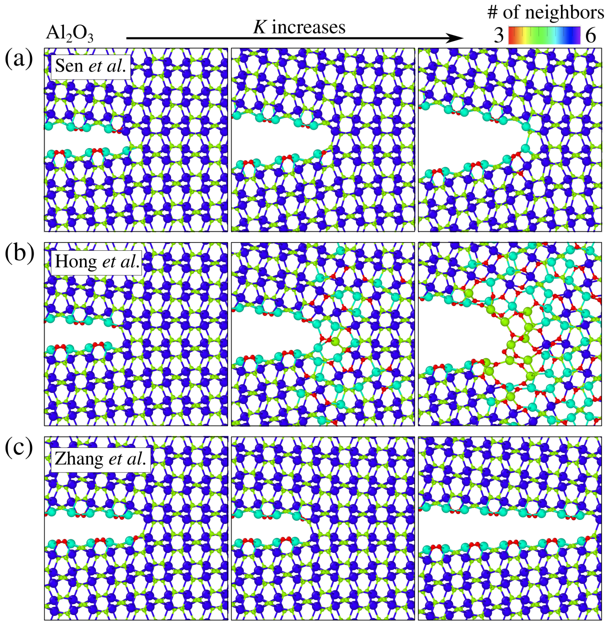

Figure S2: Crack tip evolution for corundum using different ReaxFF parameter sets: (a) Sen et al. [6], (b) Hong et al. [7] and (c) Zhang et al. [8]. Atoms are colored by the number of first neighbors.

Table S2: Comparison of lattice constants and bulk modulus for corundum for DFT and different ReaxFF parametrizations.

Method	a/b (Å)	c (Å)	B (GPa)
DFT	4.81	13.12	231
Sen et al.	4.90	13.14	234
Hong et al.	4.84	13.04	253
Zhang et al.	4.76	13.16	214

ture behavior and reasonable elasticity as indicated by Table S1. Therefore, our crack tip investigations only focused on corundum using the ReaxFF parameter set of Zhang et al.

S1.2 Free Energy of Dissolution

As the next step of our study, we computed the free energy path of Al dissolution using the chosen ReaxFF version and we found an exothermic behavior as can be seen in Figure S3. As it is well known, corundum is highly insoluble in water, which means that an increase in the free energy of such process should increase. Previous investigations of metal oxide dissolution through metadynamics have used either the distance of atom from surface $d-d_0$ [9, 10] or the coordination number of the target atom with oxygen atoms in the surroundings, especially from belonging to liquid water [11, 12, 13, 14, 15]. Using the chosen ReaxFF parameter set from the previous subsection, we performed constrained molecular dynamics of the dissolution process via Umbrella Sampling with LAMMPS [16] integrated with PLUMED [17], where the free energy path was sampled by Weighted Histogram Analysis Method (WHAM) [18]. For our purposes, $d-d_0$ seemed to be more appropriate since for sufficiently long runs, the solvation shell of the dissolved target ion is $Al^{3+}(H_2O)_6$, which is known to be the most stable one, which was revealed by our initial tests with ReaxFF as shown in Figure S3(a). When one chooses CN(Al-O$_w$), the free energy surface presents notable minima for integer values of the reaction coordinate corresponding to attachment of an additional water molecule (or hydroxyl group), as can be seen in Figure S3(b). The target atom can be fully detached as $Al^{3+}(OH^-)_3H_2O$, where it is expected to be a transient state which should transit to the predominant 6-fold coordinated ion [19]. Such discussion justify the collective variable of distance from surface.

Due to the lack of force fields appropriate for all scenarios we needed, namely brittle fracture and dissolution, we employed Density Functional Theory as the method of choice for the studies of dissolution from flat surfaces for both hematite and corundum, so that we have them on the same level of accuracy. CP2K software was chosen due to its greater efficiency/accuracy trade-offs and its larger applicability for simulations involving liquids. It is also the most widely used DFT code for enhanced-sampling molecular dynamics simulations, such as metadynamics, which is more suitable for a multiprocess reaction as atom dissolution with several atoms and bond-breaking events involved, making methods such as Nudged-Elastic Band inappropriate.

Before doing metadynamics, unbiased *ab initio* molecular dynamics during 2 ps were performed as a pre-equilibration step, where we monitored time series of the reaction coordinate $d-d_0$. It fluctuates around the initial atomic position with standard deviation of nearly 0.1 Å, which justifies the use of a Gaussian height. Figure S4 shows the free energy path collected at specific time intervals to show the convergence of the free energy path used in the main text.

Fracture energies of hematite are previously reported using PBE+U [20, 21] and are shown in Table S3. Differences found from previous works can be attributed to the use of different software, such as the Hubbard correction, which is applied in another way than CP2K. To our knowledge, free energy barriers for hematite and corundum were not found in the literature, but similar values were found in the context of mineral dissolution for iron and aluminum *hydr*oxides, such as goethite [9] (up to 3.38 eV) and gibbsite [11, 15] (up to 2.7 eV), respectively.

Table S3: Fracture energies ($R=2\gamma$) in eV/Å^2 corresponding to identical surface terminations of hematite as obtained by our results and other computational studies in the literature.

Reference	(001)	(012)	(110)
Liao et al.	0.255	0.185	not reported
Zhang et al.	0.223	0.176	0.190
This work	0.286	0.220	0.242

In order to improve clarity, a flowchart summarizing the methodology employed in each part of the work is presented in Figure S5.

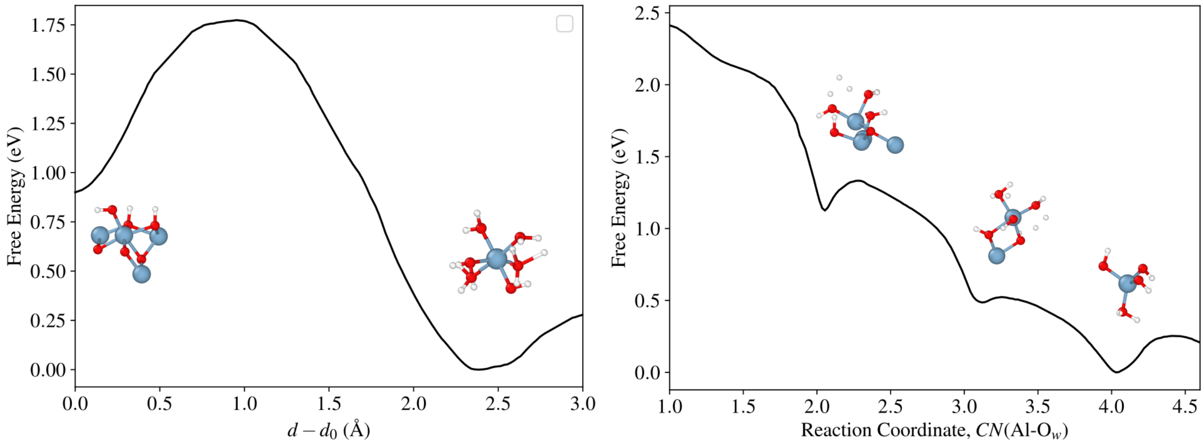

Figure S3: Free energy of dissolution computed with ReaxFF of Zhang *et. al.* [8] using (a) the atom distance from surface and (b) coordination number of target Al atom with oxygen from water as reaction coordinate. Neighborhood of the target atom is shown on the insets.

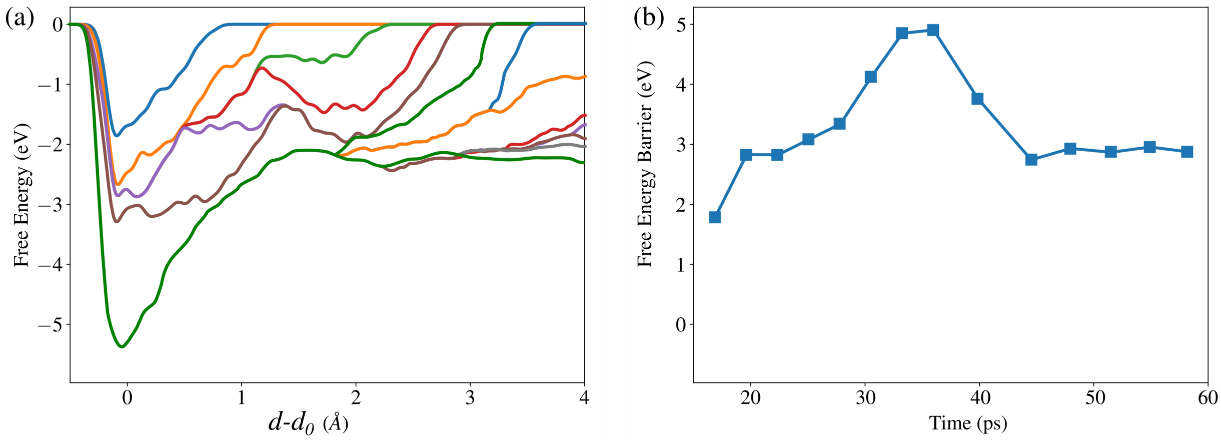

Figure S4: Convergence of the (a) free energy path and (b) the free energy barrier given by the difference between the final and initial state, computed by *ab initio* metadynamics.

S2 Basal Cleavage of Corundum

Crack propagation in α-Al_2O_3 presents a known instability along the basal plane (001) (see Figure S6(a) and (b)) as observed in early experimental works [22]. According to ReaxFF calculations, we see that such effect may be a result of the crack deflection to the [012] direction, since it has lower surface energy, as shown in Figure S6(c) and (d). Besides, Figures S6(b), (e) and (f) shows that most of the strain relaxation occurs upon AlO bond breaking at the tip, which occurs after considerable crack propagation has already occurred along [012], as observed in Figure S6(d). In this sense, a cleavage along the basal plane would be precluded by crack deflection to other crystallographic orientations possessing lower surface energies.

S3 Stress Corrosion Cracking of Silica: The Case of Cristobalite

As previously mentioned, silica polymorphs can accommodate the mechanism of chemo-rupture where molecule reach bond at the crack tip as one can compare the Connolly surface plotted for a crack in corundum and cristobalite as depicted by Figure S8. One can see that, considering a water molecule to be a sphere of ~ 2.6 Åin diameter, one can construct a surface where such a probe sphere can reach the crack tip located at $x = 0$. In this sense, we can investigate the detailed process of water molecule chemisorption at a Si-O-Si bridge at the tip apex.

For such a purpose of atomistic modeling of stress corrosion cracking in silica, we adopted a crystalline model of β-cristobalite, as depicted in Figure S9(a). We studied a crack in such a crystal propagating through the (111) plane as it provides a simple structure. As the initial state we considered a crack where all dangling O atoms are fully-passivated with hydrogen and Si with OH groups, with an additional H_2O molecule near the tip (Figure S9(b)). We considered two possible scenarios: (i) the tip bond can break without the assistance of molecule (State B), which we call it as "dry" rupture, or (ii) dissociative chemisorption may take place (State C). Thermodynamic equilibrium can be reached when the energy of initial and final states is identical, which is shown for both AB and AC transitions in Figure S9. Such points are taken as reference to analyse

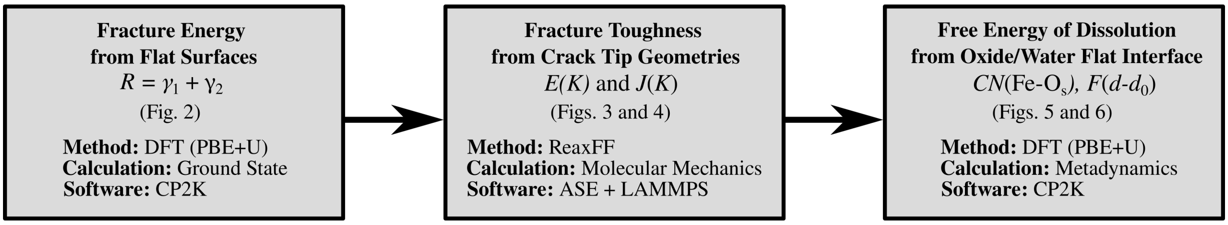

Figure S5: Flowchart diagram summarizing the methodology used in this work corresponding to each aspect investigated alongside the method, type of calculation, and software used.

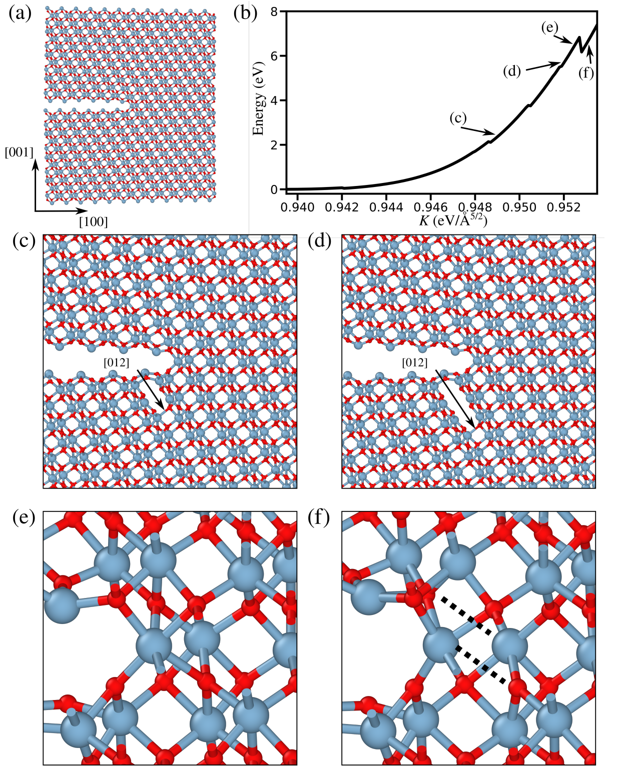

Figure S6: (a) Crack along (001) plane with Al termination. (b) Energy profile as a function of K. Panels (c) and (d) show the progression of fracture propagation, where it initially deflects along the (012) plane. Only at larger loads ($K \sim 0.952$ eV/Å$^{5/2}$), is that the bond breaking at the crack tip occur from, and intact tip shown in (e) to the breaking of Al-O bonds shown in the dashed lines (f).

how crack propagation velocity changes with applied load K. Such analysis is provided in Figure S10.

Nudge-Elastic Band (NEB) calculations were performed to obtain the activation energies for crack extension and were calculated by the improved-tangent algorithm [23] of the Nudged-Elastic Band (NEB) method as implemented by the Atomic Simulation Environment (ASE) [24]. For the systems with dry rupture, it was found that the use 21 replicas were adequate to sample the minimum energy path, as shown in a previous work [25]. As highlighted in Figure S9(b), the chemo-rupture process can be divided into two steps: (i) transport of molecules through OH terminations overcoming steric repulsion and (ii) chemisorption and dissociation at the tip. We performed separate NEB calculations for each part of the process, using 9 replicas each. For small loads, one can see that reaction at the tip is still thermodynamically unfavorable, as seen in Figure S10 with formidable barrier of 2 eV. For comparison, previous works of stress corrosion cracking of silica nanorods found a barrier for chemorupture with no applied stress of nearly 0.8 eV using semi-empirical quantum chemistry [26]. We expect that such a smaller barrier may be a consequence of the absence of steric repulsion that usually occurs inside a more realist crack tip geometry, as our study suggests. Activation energies of chemo-rupture can be dramatically reduced in the load range of thermodynamic equilibrium of a dry rupture (inset of Figure S9(c)). Crack extension without molecule assistance can also be activated by barriers as large as 2 eV, whereas chemo-rupture is favored by becoming nearly barrierless at such a load range showed in Figure S10(b). With the NEB barriers calculated for several values of K, one can see which process dominates for each load range. Note that the same K range using in (a) could not be considered for dry rupture because the final state of unpassivated broken bond would heal and recover the initial state. One can perform a linear extrapolation for barriers as $E_a(K) = E_a(0) - bK$ for both processes, which is shown in Figure S10(c). One can see that at K 0.54 eV/Å^2 there is a transition of most favorable process, where dry rupture becomes more favorable. From such data, one can estimate crack velocities by the usual transition state theory:

$$v = d_0 f_0 \exp\left(-\frac{E_a}{k_B T}\right), \tag{S1}$$

where d_0 is the distance between each bond tip (4.5 Åfor crack along (111)) and f_0 is the attempt frequency, here estimated by $\sim 3\times10^{12}$ Hz as the frequency of the lowest energy optical modes of β-cristobalite [27]. The observed transition a more favorable dry rupture is consistent with typical $v \times K$ curves of stress corrosion cracking usually reported in experimental works [28, 22, 29, 30, 31].

As opposed to the case of water-assisted cracking, showed in the Figure 4 of the main text, where a water molecule reacts with crack tip in a non-dissociative chemisorption mechanism, silica promotes water splitting to symmetrically passivated dangling bonds as a result of crack propagation. Such mechanism dramatically reduces the necessary energy to be released to fracture as computed by the J-integral of both cases. One can see that, in the breaking point of dry rupture at $K \sim 0.54$ eV/Å$^{5/2}$, $J_c/K_c \sim 1.3$, which indicates a considerable degree of lattice trapping effect [32]. For reference, perfectly brittle materials should present $J_c/K_c = 1$, where no other mechanism is present during fracture. Such effect may be due to the fact that most of the force fields tend to overestimate the

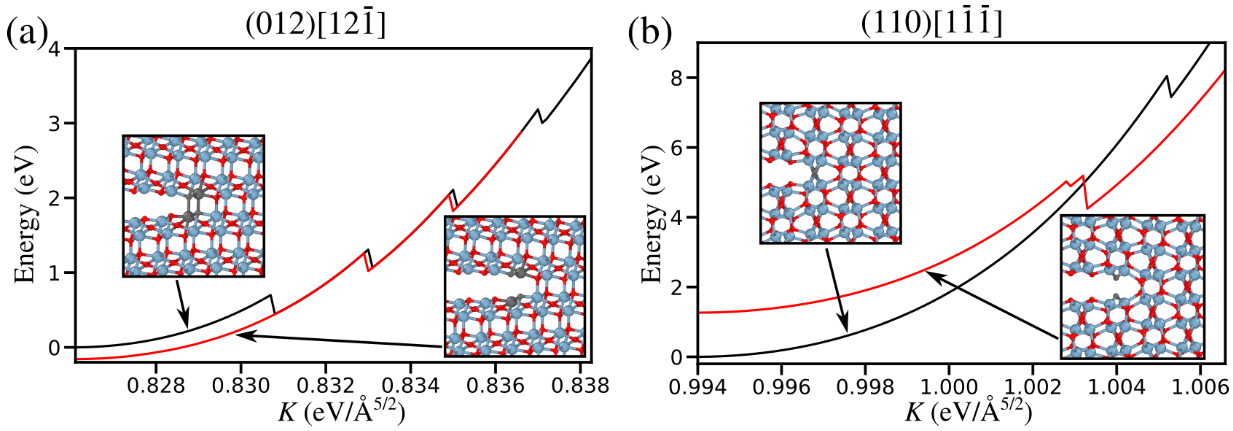

Figure S7: Energy versus K for (a) (012)[12$\bar{1}$] and (b) (110)[1$\bar{1}\bar{1}$] cracks, where the state referring to each curve is indicated on the insets by arrows.

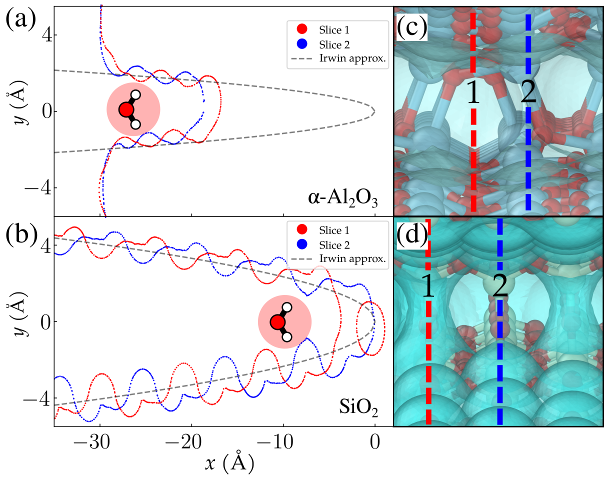

Figure S8: Accessible solvent area measured by the Connolly surface with the profiles for (a) β-cristobalite and (b) corundum for different cuts shown in the front view of the crack in (c) and (d), respectively.

lattice trapping effect of brittle fracture [25].

S4 Kinetic Model of Crack Blunting

The explicit expressions of the central quantities σ and κ in our kinetic modeling are presented, which come from the extended Charles and Hillig theory of stress corrosion cracking [33]. The stress field can be obtained by the Inglis of an elliptical crack

$$\sigma(\theta) = \sigma_\infty f(\theta), \tag{S2}$$

where σ_∞ is the uniformly applied stress far from the tip and f, is given by

$$f(\theta) = \frac{(1+\lambda)^2(1+2\cos 2\theta)-2}{2\Pi}, \tag{S3}$$

where the aspect ratio of the elliptical cavity is given by $\lambda = b/a$, and Π is

$$\Pi = \sin^2\theta + \lambda^2\cos^2\theta, \tag{S4}$$

with $g(\theta) = \lambda\Pi^{3/2}$. From the expressions above, and knowing some fundamental constants of the given material, we can obtain the curves plotted in Figure 7 in the main text.

References

[1] Huang S, Zhang S, Belytschko T, Terdalkar SS, Zhu T. Mechanics of nanocrack: Fracture, dislocation emission, and amorphization. Journal of the Mechanics and Physics of Solids. 2009 May;57(5):840-50. Available from: https://www.sciencedirect.com/science/article/pii/S0022509609000088.

[2] Lawn B. Fracture of Brittle Solids. 2nd ed. Cambridge Solid State Science Series. Cambridge: Cambridge University Press; 1993. Available from: https://www.cambridge.org/core/books/fracture-of-brittle-solids/B1EC1413BDBA1DCF49E1665D4B0A20F3.

[3] Aryanpour M, van Duin ACT, Kubicki JD. Development of a Reactive Force Field for Iron-Oxyhydroxide Systems. The Journal of Physical Chemistry A. 2010 Jun;114(21):6298-307. Publisher: American Chemical Society. Available from: https://doi.org/10.1021/jp101332k.

[4] Shin YK, Kwak H, Vasenkov AV, Sengupta D, van Duin ACT. Development of a ReaxFF Reactive Force Field for Fe/Cr/O/S and Application to Oxidation of Butane over a Pyrite-Covered Cr2O3 Catalyst. ACS Catalysis. 2015 Dec;5(12):7226-36. Publisher: American Chemical Society. Available from: https://doi.org/10.1021/acscatal.5b01766.

[5] Huang Y, Hu C, Xiao Z, Gao N, Wang Q, Liu Z, et al. Atomic insight into iron corrosion exposed to supercritical water environment with an improved Fe-H2O reactive force field. Applied Surface Science. 2022 Apr;580:152300. Available from: https://www.sciencedirect.com/science/article/pii/S0169433221033249.

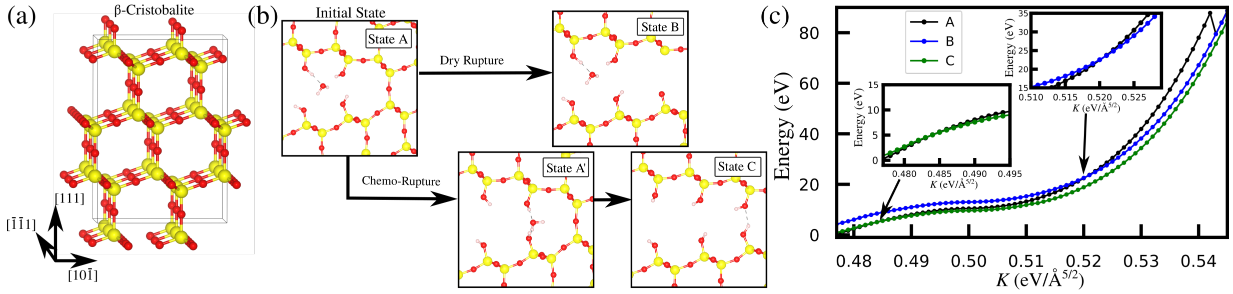

Figure S9: Energetics of a sharp crack in a crystalline silica model. (a) The unit cell of the β-cristobalite is used as the silica polymorph with the [111] surface along the z-axis, which is replicated to obtain the crack system. (b) Description of the two processes considered: (i) rupture, where the crack tip bond breaks due to the applied load only (without immediate molecule chemisorption) and (ii) chemo-rupture where bond breaking takes place by reaction with the nearby water molecule. (c) Energy curves for each state (A, B, and C) showing crossover points where one state begins to be more favorable than others.

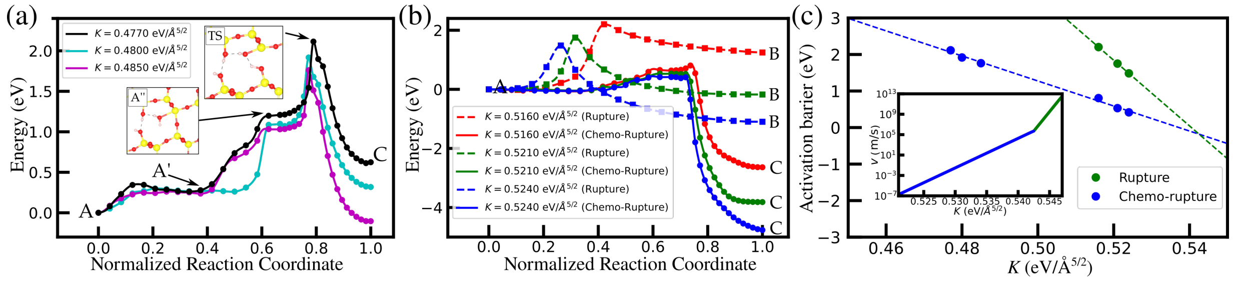

Figure S10: Kinetics of a sharp crack in cristobalite. (a) Energy barriers obtained with NEB method for water reaction at the crack tip for K values around the crossover points shown in the inset on Figure S9(c) between states A and C. (b) Energy barriers around the crossover points between states A and B, where we compare the minimum energy paths for rupture and chemo-rupture, where their activation energies are shown in (c), where we can see that at $K \sim 0.54$ eV/Å$^{5/2}$ rupture becomes the most probable process instead of reaction at the tip. The crack propagation velocity in the inset.

[6] Sen FG, Qi Y, van Duin ACT, Alpas AT. Oxidation induced softening in Al nanowires. Applied Physics Letters. 2013 Feb;102(5):051912. Available from: https://doi.org/10.1063/1.4790181.

[7] Hong S, van Duin ACT. Atomistic-Scale Analysis of Carbon Coating and Its Effect on the Oxidation of Aluminum Nanoparticles by ReaxFF-Molecular Dynamics Simulations. The Journal of Physical Chemistry C. 2016 May;120(17):9464-74. Publisher: American Chemical Society. Available from: https://doi.org/10.1021/acs.jpcc.6b00786.

[8] Zhang Y, Liu X, van Duin ACT, Lu X, Meijer EJ. Development and validation of a general-purpose ReaxFF reactive force field for earth material modeling. The Journal of Chemical Physics. 2024 Mar;160(9):094103. Available from: https://doi.org/10.1063/5.0194486.

[9] Klyukin K, Rosso KM, Alexandrov V. Iron Dissolution from Goethite (α-FeOOH) Surfaces in Water by Ab Initio Enhanced Free-Energy Simulations. The Journal of Physical Chemistry C. 2018 Jul;122(28):16086-91. Publisher: American Chemical Society. Available from: https://doi.org/10.1021/acs.jpcc.8b03743.

[10] Raman AS, Vojvodic A. Providing Atomistic Insights into the Dissolution of Rutile Oxides in Electrocatalytic Water Splitting. The Journal of Physical Chemistry C. 2022 Jan;126(2):922-32. Publisher: American Chemical Society. Available from: https://doi.org/10.1021/acs.jpcc.1c08737.

[11] Shen Z, Kerisit SN, Stack AG, Rosso KM. Free-Energy Landscape of the Dissolution of Gibbsite at High pH. The Journal of Physical Chemistry Letters. 2018 Apr;9(7):1809-14. Publisher: American Chemical Society. Available from: https://doi.org/10.1021/acs.jpclett.8b00484.

[12] Réocreux R, Girel E, Clabaut P, Tuel A, Besson M, Chaumonnot A, et al. Reactivity of shape-controlled crystals and metadynamics simulations locate the weak spots of alumina in water. Nature Communications. 2019 Jul;10(1):3139. Publisher: Nature Publishing Group. Available from: https://www.nature.com/articles/s41467-019-10981-9.

[13] Schliemann R, Churakov SV. Atomic scale mechanism of clay minerals dissolution revealed by ab initio simulations. Geochimica et Cosmochimica Acta. 2021 Jan;293:438-60. Available from: https://www.sciencedirect.com/science/article/pii/S0016703720306608.

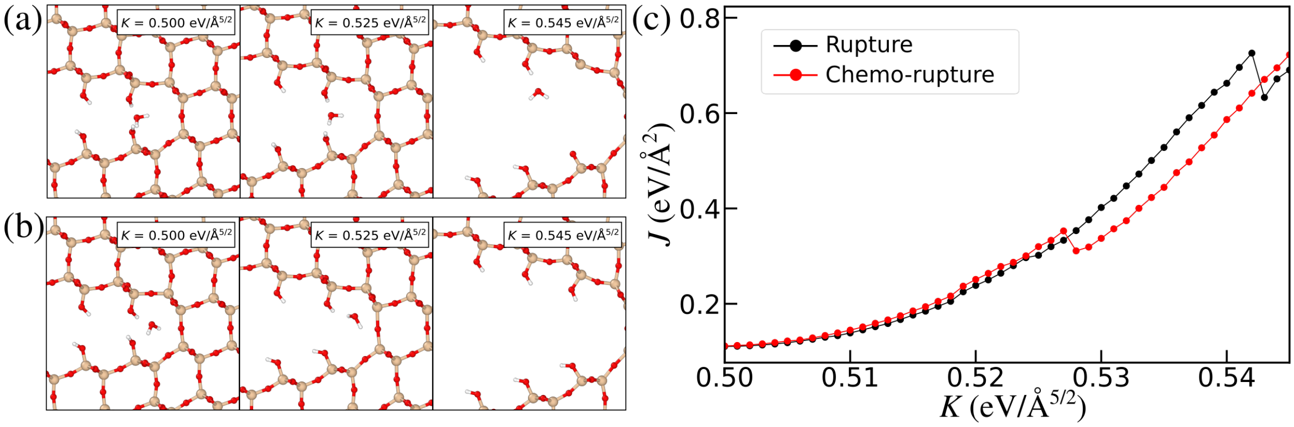

Figure S11: J-integral calculation for the rupture and chemo-rupture processes involved in the fracture of cristobalite with snapshots for different loads (indicated) shown in (a) and (b), respectively. (c) J versus K curve for both cases, showing an early decrease of J_c upon molecule chemisorption at the tip.

[14] Liu M, Ruiz Pestana L. Mechanisms and energetics of calcium aluminosilicate glass dissolution through ab initio molecular dynamics-metadynamics simulations. npj Materials Degradation. 2024 Mar;8(1):1-9. Publisher: Nature Publishing Group. Available from: https://www.nature.com/articles/s41529-024-00445-x.

[15] Guo Q, Pouvreau M, Rosso KM, Clark AE. Mechanisms of dissolution from gibbsite step edges elucidated by ab initio molecular dynamics with enhanced sampling. Geochimica et Cosmochimica Acta. 2024 Feb;366:201-9. Available from: https://www.sciencedirect.com/science/article/pii/S0016703723005690.

[16] Thompson AP, Aktulga HM, Berger R, Bolintineanu DS, Brown WM, Crozier PS, et al. LAMMPS - a flexible simulation tool for particle-based materials modeling at the atomic, meso, and continuum scales. Computer Physics Communications. 2022 Feb;271:108171. Available from: https://www.sciencedirect.com/science/article/pii/S0010465521002836.

[17] Tribello GA, Bonomi M, Branduardi D, Camilloni C, Bussi G. PLUMED 2: New feathers for an old bird. Computer Physics Communications. 2014 Feb;185(2):604-13. Available from: https://www.sciencedirect.com/science/article/pii/S0010465513003196.

[18] Grossfield A. WHAM – Grossfield Lab;. Available from: http://membrane.urmc.rochester.edu/?page_id=126.

[19] Lubin MI, Bylaska EJ, Weare JH. Ab initio molecular dynamics simulations of aluminum ion solvation in water clusters. Chemical Physics Letters. 2000 Jun;322(6):447-53. Available from: https://www.sciencedirect.com/science/article/pii/S0009261400004346.

[20] Liao P, Carter EA. Ab initio DFT + U predictions of tensile properties of iron oxides. Journal of Materials Chemistry. 2010 Aug;20(32):6703-19. Publisher: The Royal Society of Chemistry. Available from: https://pubs.rsc.org/en/content/articlelanding/2010/jm/c0jm01199a.

[21] Zhang H, Sun W, Xie X, He J, Zhang C. Insights into the Fracture Nature of Hematite from First Principles DFT Calculations. ACS Omega. 2023 Mar;8(9):8248-55. Publisher: American Chemical Society. Available from: https://doi.org/10.1021/acsomega.2c06101.

[22] Wiederhorn SM, Fuller ER, Thomson R. Micromechanisms of crack growth in ceramics and glasses in corrosive environments. Metal Science. 1980 Aug;14(8-9):450-8. Publisher: Taylor & Francis. Available from: https://www.tandfonline.com/doi/abs/10.1179/msc.1980.14.8-9.450.

[23] Henkelman G, Jónsson H. Improved tangent estimate in the nudged elastic band method for finding minimum energy paths and saddle points. The Journal of Chemical Physics. 2000 Dec;113(22):9978-85. Available from: https://doi.org/10.1063/1.1323224.

[24] Larsen AH, Mortensen JJ, Blomqvist J, Castelli IE, Christensen R, Dułak M, et al. The atomic simulation environment—a Python library for working with atoms. Journal of Physics: Condensed Matter. 2017 Jun;29(27):273002. Publisher: IOP Publishing. Available from: https://dx.doi.org/10.1088/1361-648X/aa680e.

[25] Zhang S, Zhu T, Belytschko T. Atomistic and multiscale analyses of brittle fracture in crystal lattices. Physical Review B. 2007 Sep;76(9):094114. Publisher: American Physical Society. Available from: https://link.aps.org/doi/10.1103/PhysRevB.76.094114.

[26] Zhu T, Li J, Lin X, Yip S. Stress-dependent molecular pathways of silica–water reaction. Journal of the Mechanics and Physics of Solids.

2005 Jul;53(7):1597-623. Available from: https://www.sciencedirect.com/science/article/pii/S0022509605000384.

[27] Feng SQ, Zang HP, Wang YQ, Cheng XL, Yue JS. Ab initio investigation of photoinduced non-thermal phase transition in β-cristobalite. Chinese Physics B. 2015 Nov;25(1):016701. Publisher: IOP Publishing. Available from: https://dx.doi.org/10.1088/1674-1056/25/1/016701.

[28] Wiederhorn SM. Moisture assisted crack growth in ceramics. International Journal of Fracture Mechanics. 1968 Jun;4(2):171-7. Available from: https://doi.org/10.1007/BF00188945.

[29] Michalske TA, Freiman SW. A molecular interpretation of stress corrosion in silica. Nature. 1982 Feb;295(5849):511-2. Publisher: Nature Publishing Group. Available from: https://www.nature.com/articles/295511a0.

[30] Michalske TA, Bunker BC, Freiman SW. Stress Corrosion of Ionic and Mixed Ionic/Covalent Solids. Journal of the American Ceramic Society. 1986;69(10):721-4. _eprint: https://onlinelibrary.wiley.com/doi/pdf/10.1111/j.1151-2916.1986.tb07332.x. Available from: https://onlinelibrary.wiley.com/doi/abs/10.1111/j.1151-2916.1986.tb07332.x.

[31] Michalske TA, Bunker BC. Steric Effects in Stress Corrosion Fracture of Glass. Journal of the American Ceramic Society. 1987;70(10):780-4. _eprint: https://onlinelibrary.wiley.com/doi/pdf/10.1111/j.1151-2916.1987.tb04879.x. Available from: https://onlinelibrary.wiley.com/doi/abs/10.1111/j.1151-2916.1987.tb04879.x.

[32] Meng F, Chen C, Song J. Lattice trapping and crack decohesion in graphene. Carbon. 2017 May;116:33-9. Available from: https://www.sciencedirect.com/science/article/pii/S000862231730101X.

[33] Chuang Tj, Fuller Jr ER. Extended Charles–Hillig Theory for Stress Corrosion Cracking of Glass. Journal of the American Ceramic Society. 1992;75(3):540-5. _eprint: https://onlinelibrary.wiley.com/doi/pdf/10.1111/j.1151-2916.1992.tb07839.x. Available from: https://onlinelibrary.wiley.com/doi/abs/10.1111/j.1151-2916.1992.tb07839.x.
