# Supplementary figures and images for: Ab Initio Molecular Dynamics Insights into Stress Corrosion Cracking and Dissolution of Metal Oxides
Source: Materials (Basel). 2025 Jan 24;18(3):538. doi: 10.3390/ma18030538 (PMC11818312; doi:10.3390/ma18030538)

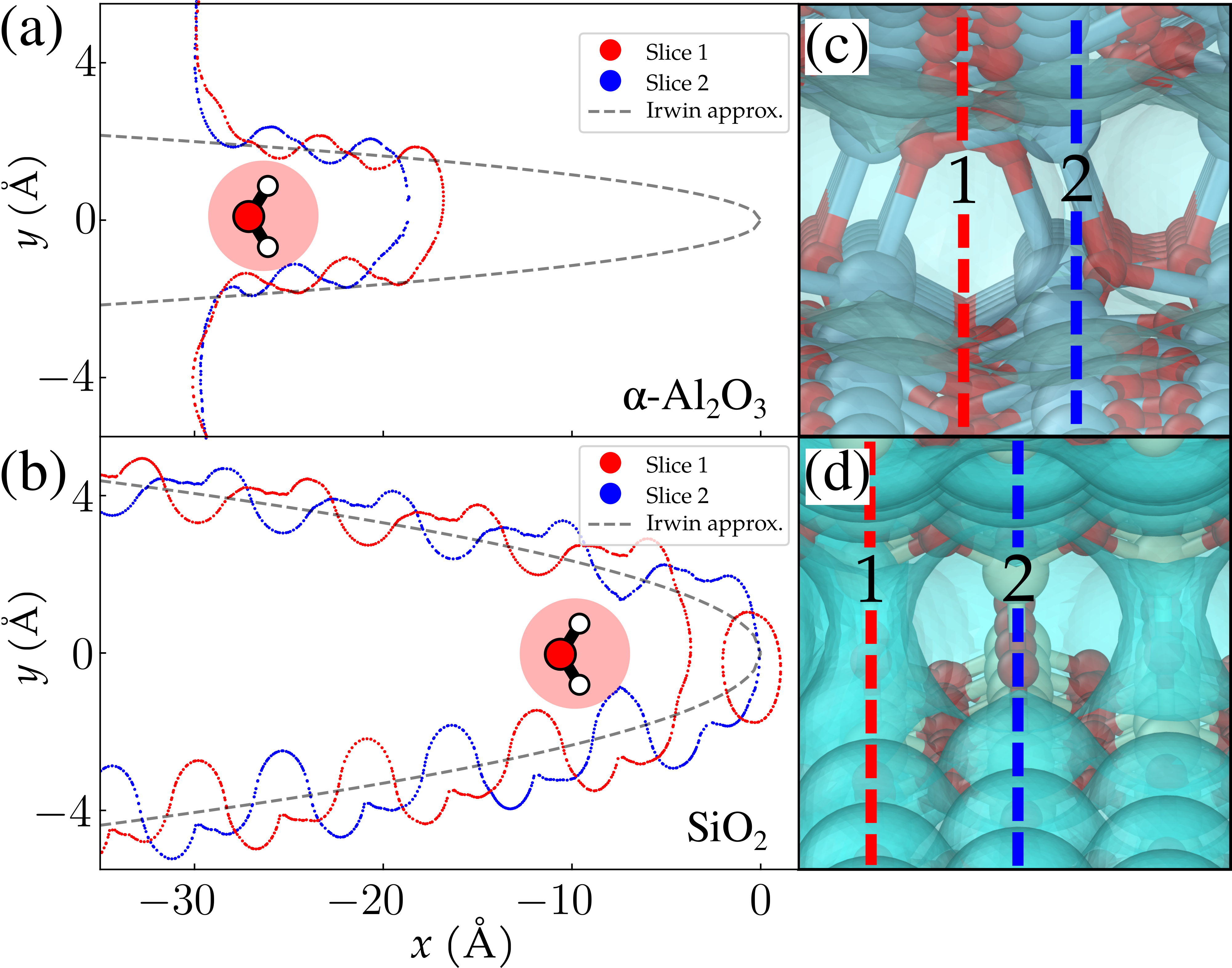

Supplement: Supplementary file 1 [file materials-18-00538-s001.zip › figures/connolly.png]

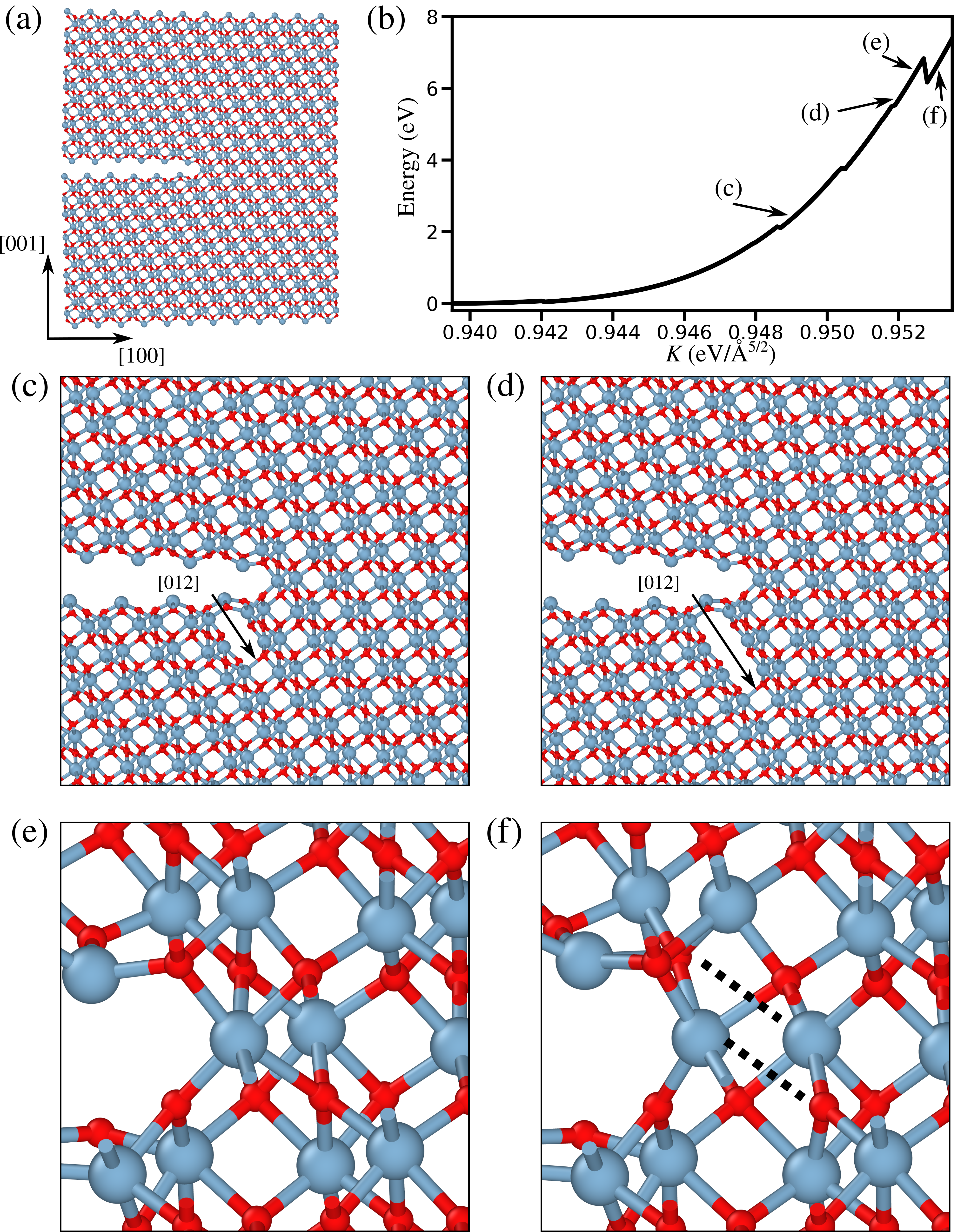

Supplement: Supplementary file 1 [file materials-18-00538-s001.zip › figures/crack-001.png]

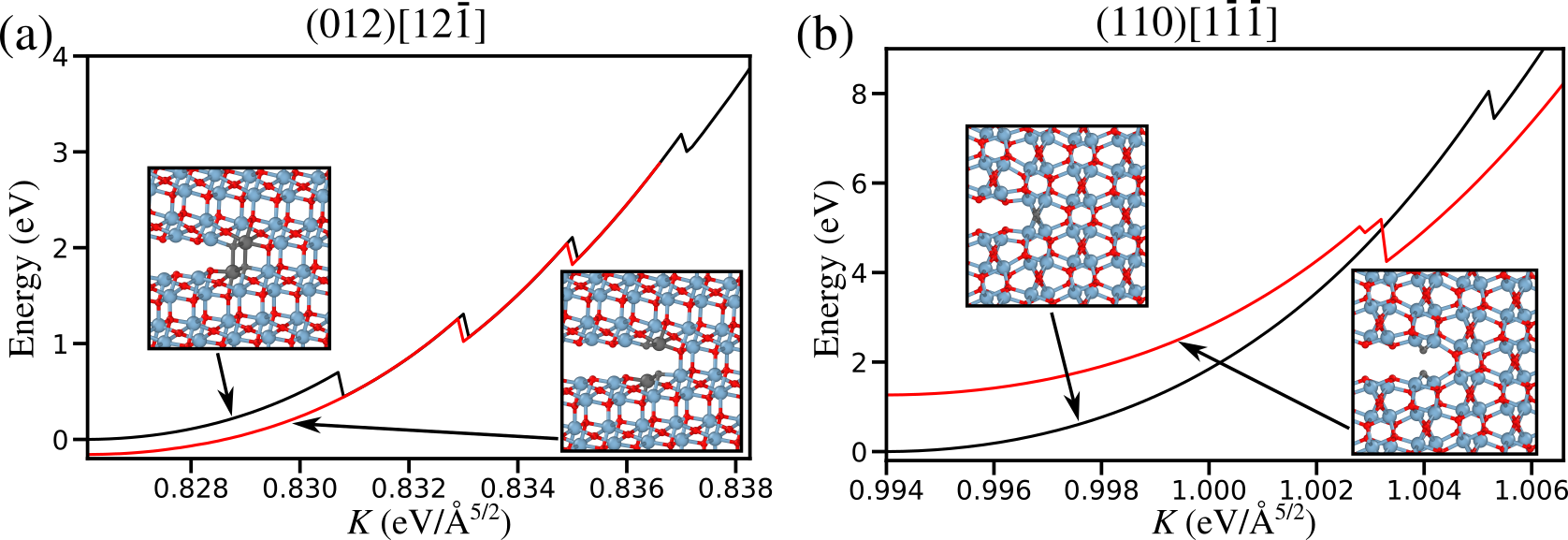

Supplement: Supplementary file 1 [file materials-18-00538-s001.zip › figures/crack-others.png]

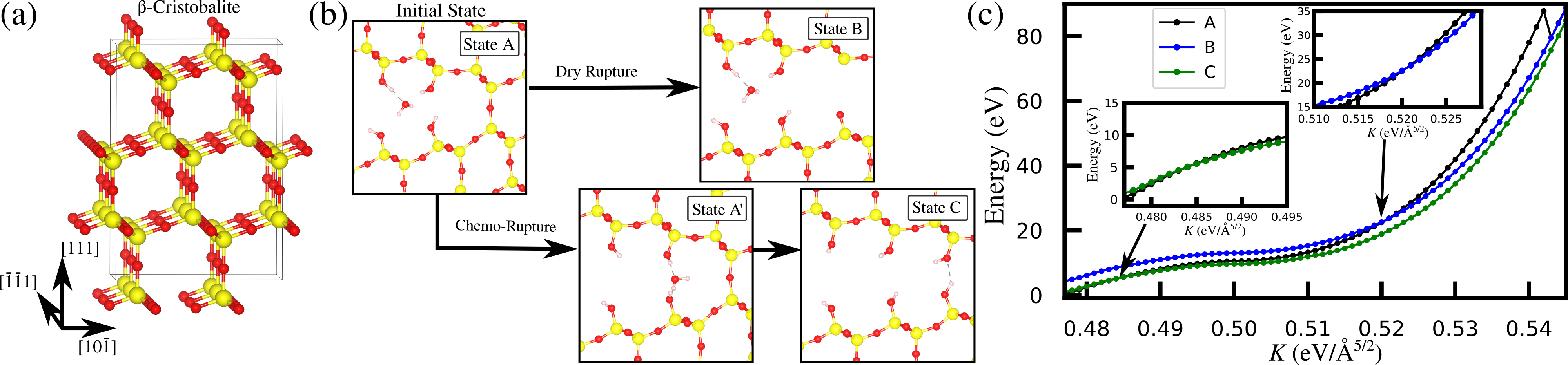

Supplement: Supplementary file 1 [file materials-18-00538-s001.zip › figures/crack-silica-1.png]

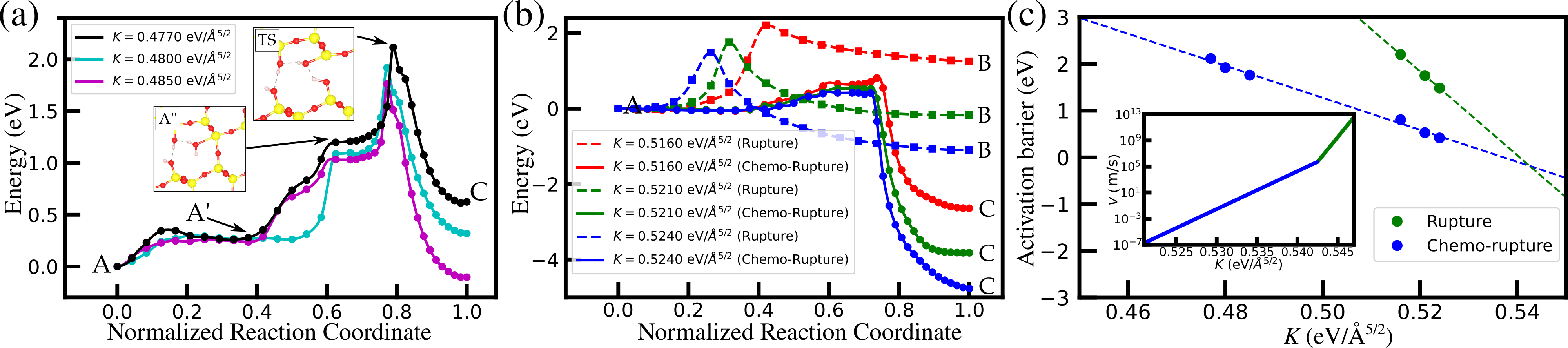

Supplement: Supplementary file 1 [file materials-18-00538-s001.zip › figures/crack-silica-2.png]

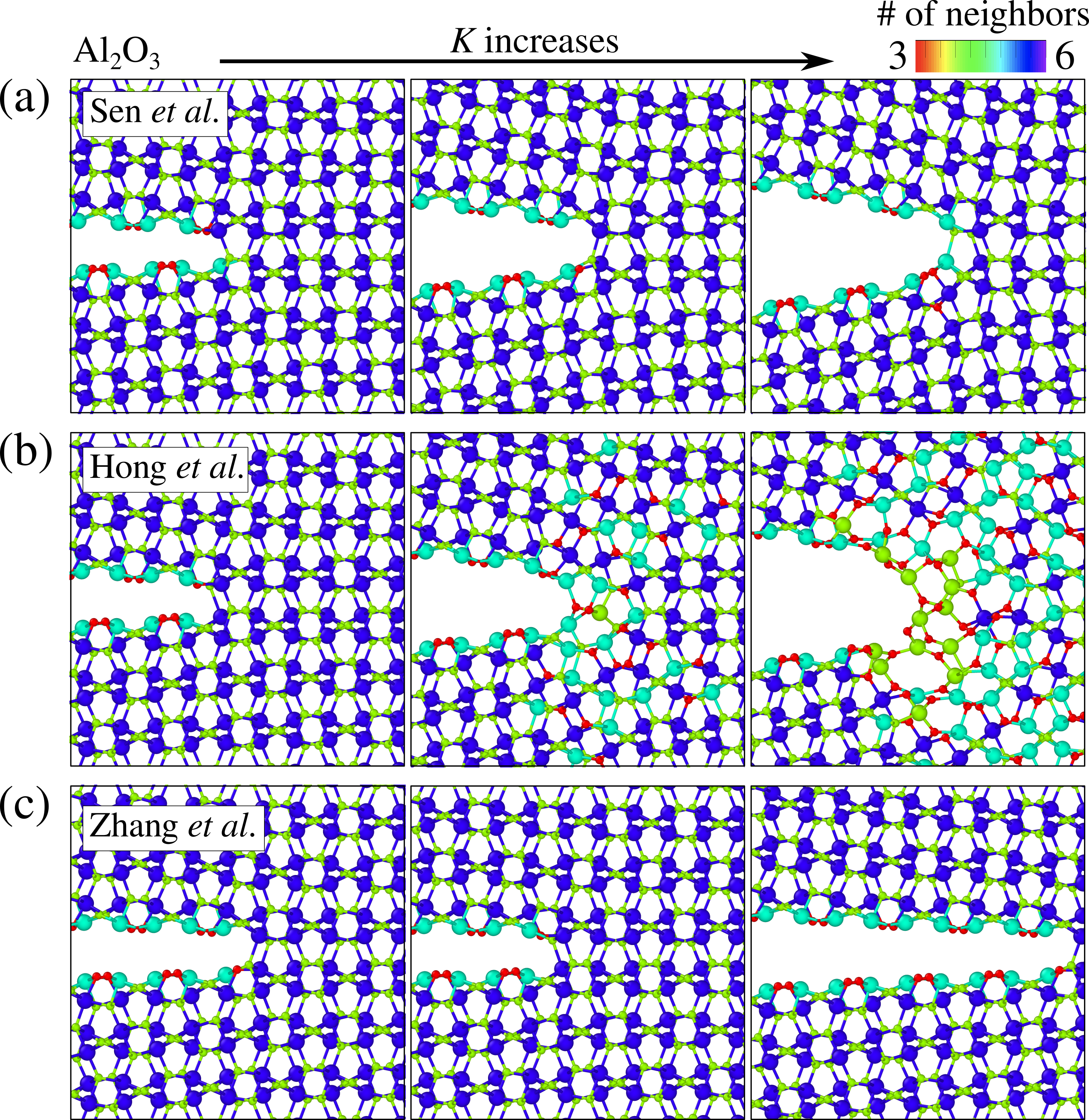

Supplement: Supplementary file 1 [file materials-18-00538-s001.zip › figures/crack-tip-al2o3.png]

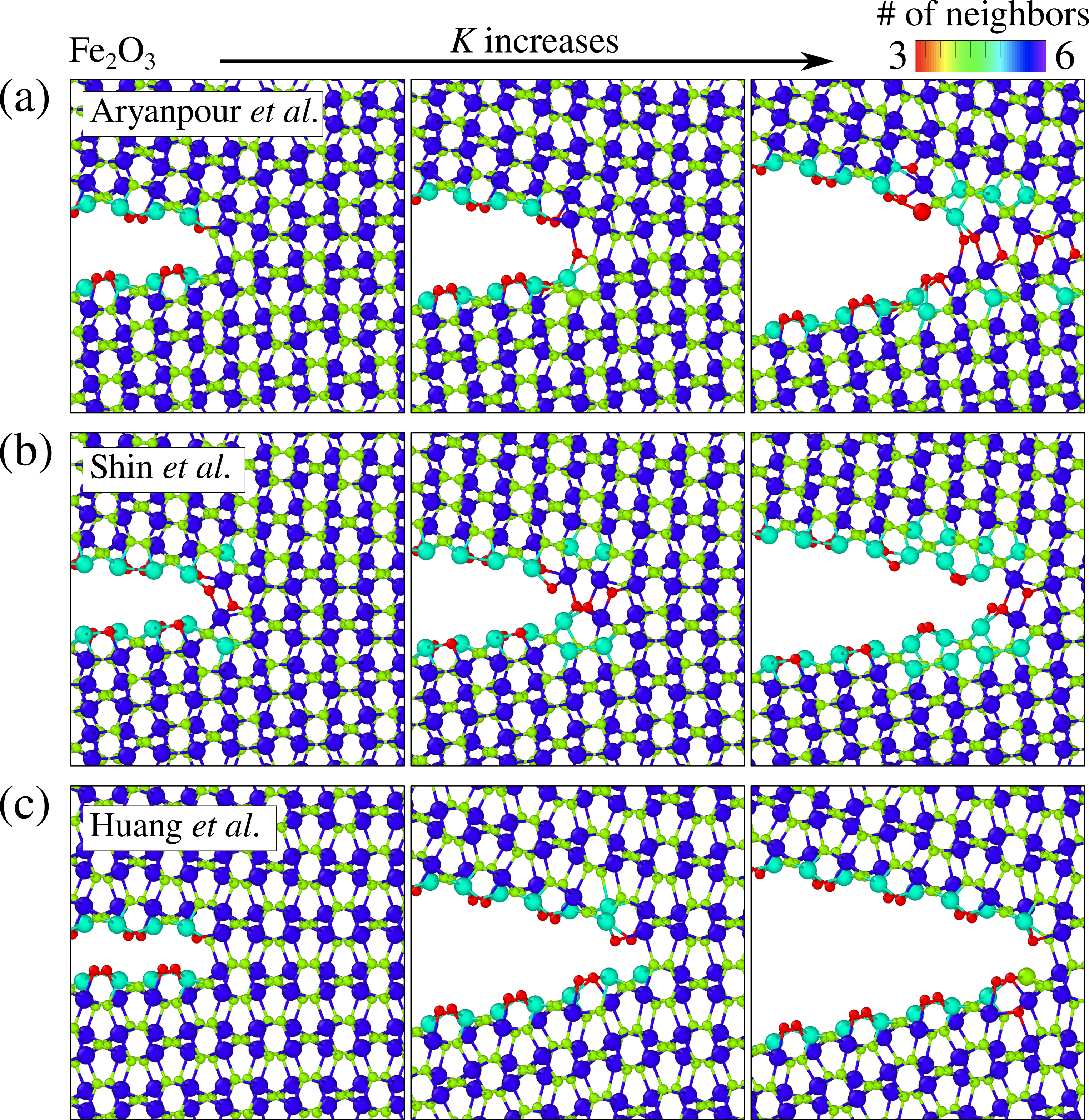

Supplement: Supplementary file 1 [file materials-18-00538-s001.zip › figures/crack-tip-fe2o3.png]

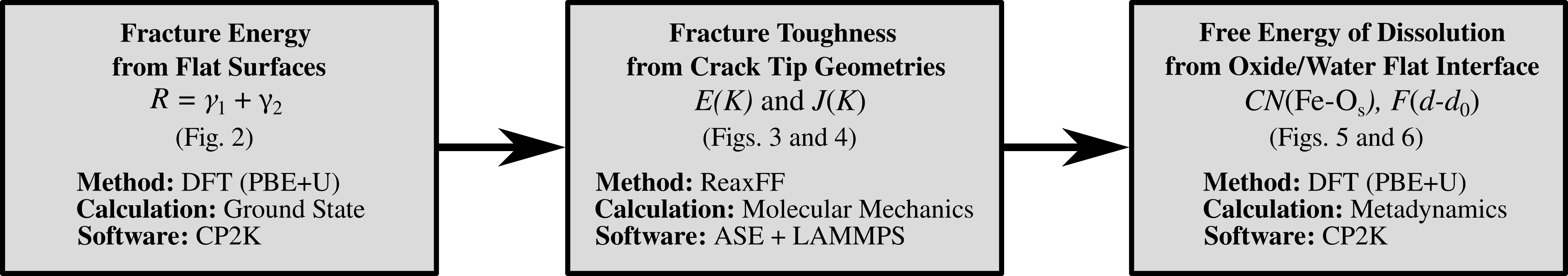

Supplement: Supplementary file 1 [file materials-18-00538-s001.zip › figures/flowchart.png]

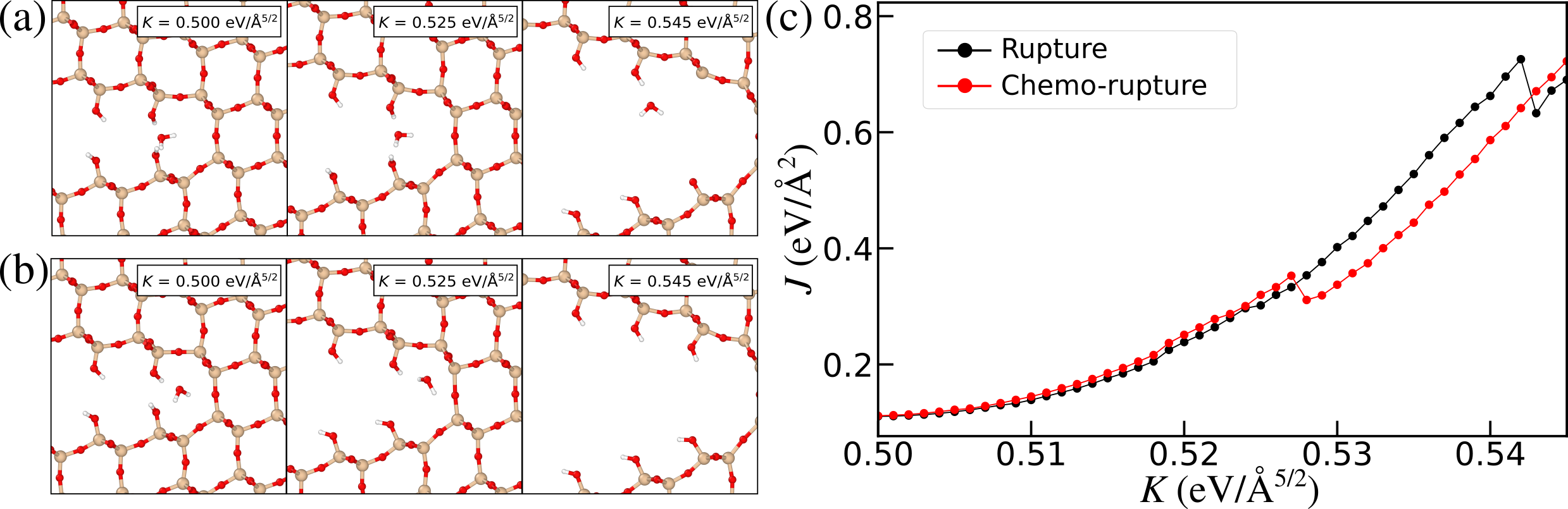

Supplement: Supplementary file 1 [file materials-18-00538-s001.zip › figures/j-silica.png]

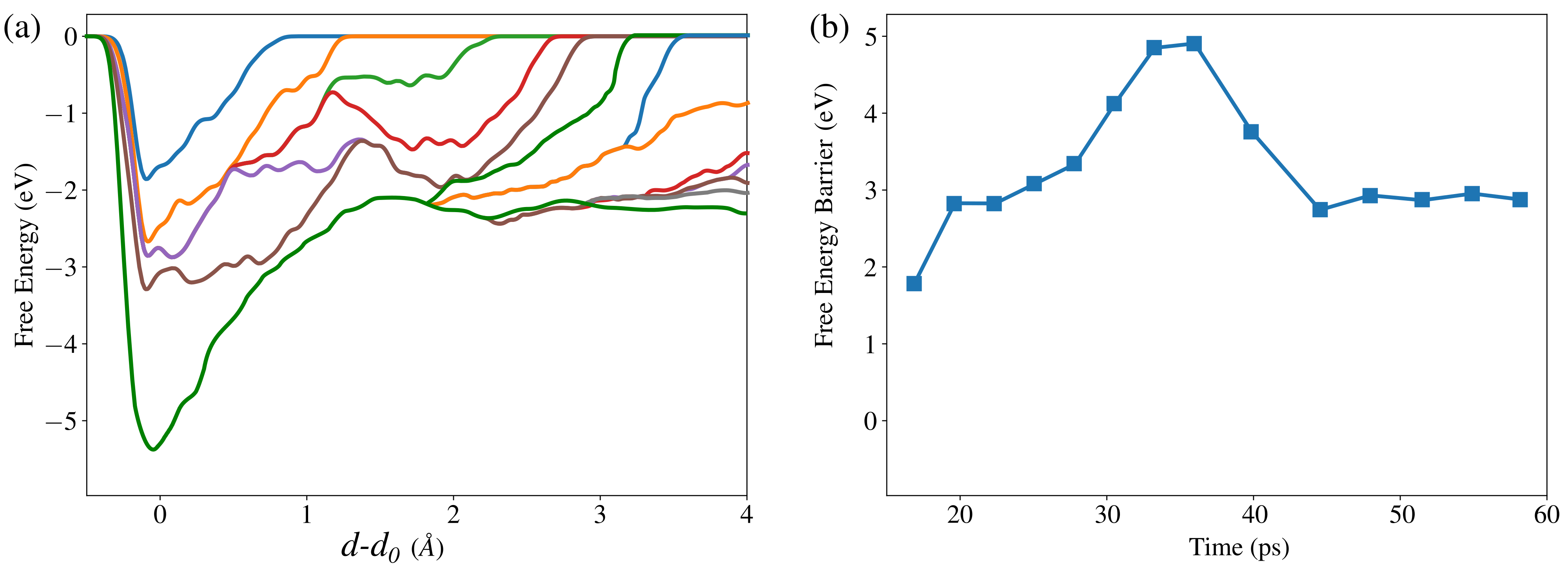

Supplement: Supplementary file 1 [file materials-18-00538-s001.zip › figures/mtd-convergence.png]

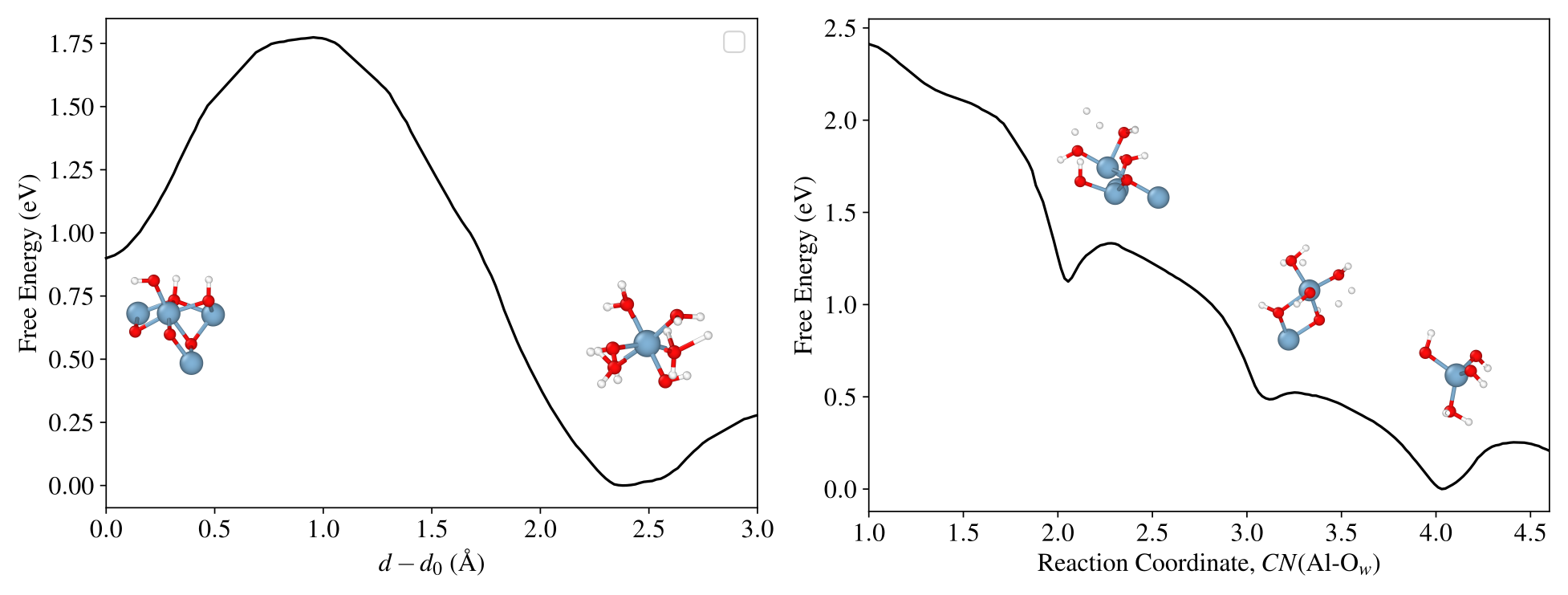

Supplement: Supplementary file 1 [file materials-18-00538-s001.zip › figures/reaxff-dissolution.png]
